# Identification of unusual peptides with new Cys frameworks in the venom of the cold-water sea anemone *Cnidopus japonicus*

**DOI:** 10.1038/s41598-017-14961-1

**Published:** 2017-11-06

**Authors:** Vladislav V. Babenko, Alexander N. Mikov, Valentin A. Manuvera, Nickolay A. Anikanov, Sergey I. Kovalchuk, Yaroslav A. Andreev, Yulia A. Logashina, Daniil A. Kornilov, Alexander I. Manolov, Nadya P. Sanamyan, Karen E. Sanamyan, Elena S. Kostryukova, Sergey A. Kozlov, Eugene V. Grishin, Vadim M. Govorun, Vassili N. Lazarev

**Affiliations:** 1 Federal Research and Clinical Centre of Physical-Chemical Medicine, Moscow, 119435 Russia; 2 Shemyakin-Ovchinnikov Institute of Bioorganic Chemistry, Moscow, 117997 Russia; 3 Moscow Institute of Physics and Technology (State University), Dolgoprudny, 141700 Russia; 4 Kamchatka Branch of Pacific Geographical Institute, Far-Eastern Branch of the Russian Academy of Sciences, Petropavlovsk-Kamchatsky, 683000 Russia; 5 Sechenov First Moscow State Medical University, Institute of Molecular Medicine, Moscow, 119991 Russia

## Abstract

Sea anemones (Actiniaria) are intensely popular objects of study in venomics. Order Actiniaria includes more than 1,000 species, thus presenting almost unlimited opportunities for the discovery of novel biologically active molecules. The venoms of cold-water sea anemones are studied far less than the venoms of tropical sea anemones. In this work, we analysed the molecular venom composition of the cold-water sea anemone *Cnidopus japonicus*. Two sets of NGS data from two species revealed molecules belonging to a variety of structural classes, including neurotoxins, toxin-like molecules, linear polypeptides (Cys-free), enzymes, and cytolytics. High-throughput proteomic analyses identified 27 compounds that were present in the venoms. Some of the toxin-like polypeptides exhibited novel Cys frameworks. To characterise their function in the venom, we heterologously expressed 3 polypeptides with unusual Cys frameworks (designated CjTL7, CjTL8, and AnmTx Cj 1c-1) in *E. coli*. Toxicity tests revealed that the CjTL8 polypeptide displays strong crustacean-specific toxicity, while AnmTx Cj 1c-1 is toxic to both crustaceans and insects. Thus, an improved NGS data analysis algorithm assisted in the identification of toxins with unusual Cys frameworks showing no homology according to BLAST. Our study shows the advantage of combining omics analysis with functional tests for active polypeptide discovery.

## Introduction

Organisms that produce venom have long been important for a number of applications. Such organisms produce a wide range of compounds with different functions (e.g., neurotoxic, haemolytic)^1,2^. These compounds are used in molecular biology, physiology, pharmacology, medicine, and the food and biotechnology industries^3–5^. At present, 2 main approaches are used to study the molecular complexity of venom components. First, the traditional approach consists of the following sequential steps: multistage fractionation of the venom into individual components, structural determination, and identification of molecular actions using different targets^6,7^. The second approach is based on a combination of next-generation sequencing (NGS) transcriptomics and proteomics^8–13^.

Although the NGS-based approach does not allow researchers to determine the biological activity of molecules, it provides comprehensive sequence information, thus helping to overcome the time and technical limitations associated with Edman degradation and shotgun MS-MS *de novo* sequencing. Using this approach, researchers can search for both new toxins and toxins that are homologous to those described previously. Large sets of possible toxin candidates derived from transcriptomics data may be validated using proteomics to determine the peptide and protein compositions of real venoms. Thus, the combination of transcriptomics and proteomics allows reductions in time and resource consumption while elucidating new polypeptides. In recent years, this approach has been popular for studying the venoms of various animals, such as spiders^14–16^, scorpions^11^, gastropods^17^, snakes^9,18^, amphibians^19^, insects^20,21^, and jellyfish^13^.

We chose a Pacific Ocean inhabitant, the cold-water sea anemone *Cnidopus japonicus* (Verrill, 1871) (also known as *Epiactis japonica*) as the object of this research. Sea anemones have long attracted the attention of scientists, both as interesting marine animals (from the perspective of classical biology) and as a source of venom (from the perspective of toxinology and biochemistry). The first sea anemone toxinology studies were conducted between 1969 and 1973^22,23^. Most sea anemones are venomous, and it is well established that their venoms contain phospholipases, cytolysins, neurotoxins, proteolytic enzymes, and protease inhibitors^24,25^.

Today, most scientific knowledge about the components of sea anemone venoms is obtained by studying warm-water (tropical) sea anemones. Cold-water sea anemone venoms are studied far less. In this work, we employed an approach based on the NGS sequencing of transcriptomes (RNA-seq) to analyse the diversity of expressed peptides and protein molecules in 2 specimens of the cold-water sea anemone *C. japonicus* and validated the results using proteomics. Moreover, we functionally characterised 3 polypeptides from *C. japonicus* venom through toxicity tests on crustaceans and insects.

## Results

### RNA-seq and de novo assembly

After cDNA library preparation, we obtained 2 independent datasets for *C. japonicus* Yellow (a yellow specimen of *Cnidopus japonicus*) and *C. japonicus* Red (a red specimen of *Cnidopus japonicus*). The cDNA libraries were sequenced using an Ion Torrent PGM sequencer with a 318 chip, to which the samples were applied in equal quantities. The results of our statistical analysis of the data are presented in Table S1.

### Analysis of the initial data

Three methods were employed to identify the sequences of the genes encoding the venom components. **Method 1** was based on the analysis of all consensus sequences (isotigs) obtained as a result of *de novo* assembly using a local BlastX algorithm. The reference database NR was used for annotation. During the next stage, all selected isotigs that were homologous to known toxins were translated using 6 reading frames in the «Predicted protein set 1» database compilation. This database was then compared with proteomics data to identify the toxins expressed in the venom, similar to the method described by Nesvizhskii *et al*.^26^ Venom samples from *C. japonicus* Yellow and *C. japonicus* Red were analysed separately.

**Figure 1:**
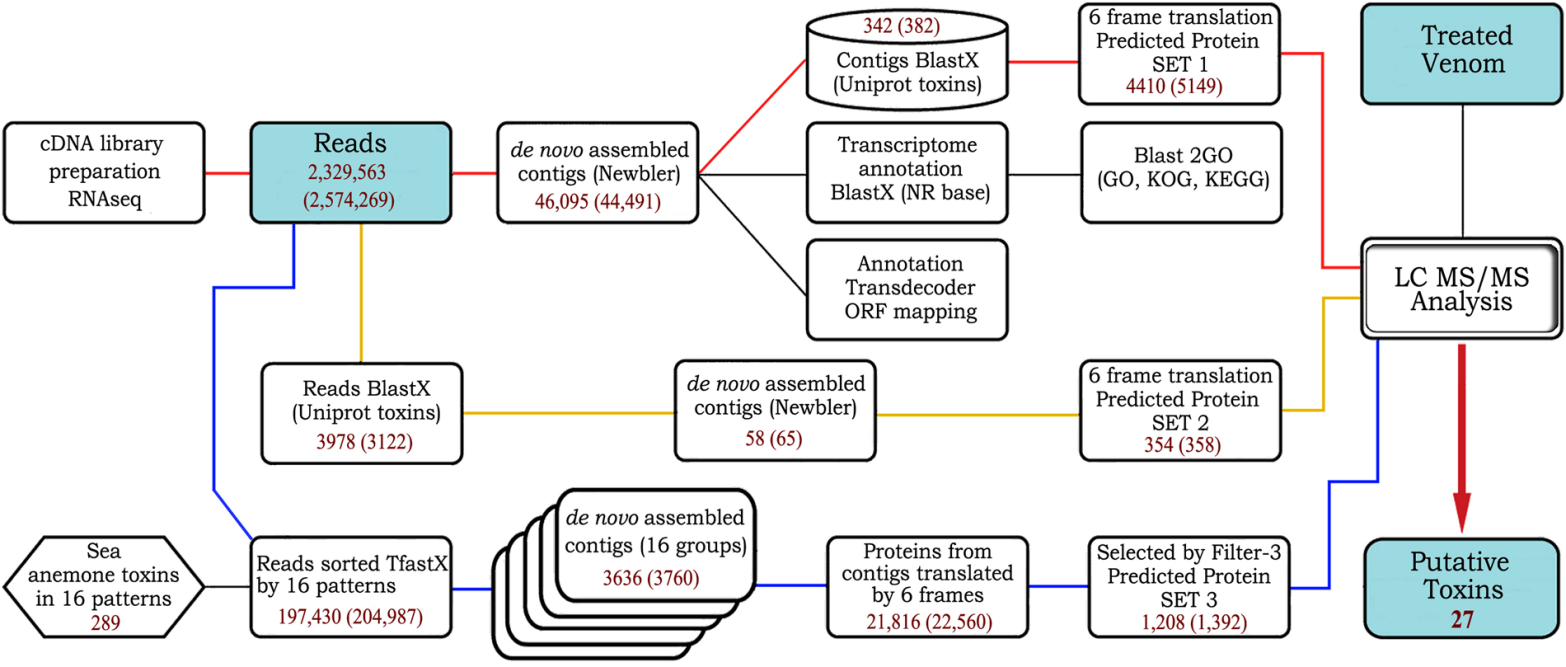
Analysis pipeline of the research implemented for *C. japonicus* Yellow (*C. japonicus* Red) samples. Method paths are coloured: red for method 1, yellow for method 2, and blue for method 3. Filter 3 includes the deletion of (**a**) very short sequences, (**b**) sequences without signal peptides, and (**c**) sequences with long repeats.

Animal venoms represent very large combinational libraries of polypeptides that differ in a number of point mutations^27^. Thus, the process of assembling contigs is prone to errors. Therefore, we developed 2 additional methods of analysis that allowed us to identify new candidates (Fig. 1).

**Method 2** was based on the elimination of raw reads (with trimmed adapter sequences) using a local BlastX algorithm. The analysis of reads, which was performed without the preliminary assembly of contigs, led to the identification of reads corresponding to toxin sequences. As a reference database, we used all toxin sequences from the UniProt animal toxin annotation program. Then, reads corresponding to animal toxins (3978 for *C. japonicus* Yellow and 3122 for *C. japonicus* Red) were assembled into contigs using Newbler. Finally, these contigs were translated using 6 reading frames in «Predicted protein set 2» for subsequent validation by proteomics.

The aim of **method 3** was to assemble transcripts related to the polypeptide toxins of sea anemones. Based on current scientific knowledge, the peptide components of sea anemone venoms were subdivided into several structural groups according to differences in the cysteine residue distribution^28,29^. In total, 16 protein sets were constructed using 289 amino acid sequences from UniProt and the translated nucleotide sequences of the toxins (see list of UniProt indicators of the sequences of the 98 toxins used in Table S2). Each set of toxins was then separately compared to the raw reads using TfastX (threshold E-value = 1.0), and reads that shared sequence similarities with toxins were subjected to further assembly. As a result, 197,430 reads for *C. japonicus* Yellow and 204,987 reads for *C. japonicus* Red were assembled into 3636 and 3760 contigs, respectively, employing CLC Genomics Workbench 7. All assembled contigs were translated using 6 reading frames, and additional filtration steps were applied, including the exclusion of ORF/precursor proteins that (1) were shorter than 50 aa, (2) contained early termination signals, (3) exhibited more than 5 amino acid repeats, and (4) lacked a signal peptide (“filter 3”). After applying filter 3, the numbers of sequences were reduced to 1208 and 1392, respectively.

**Figure 2:**
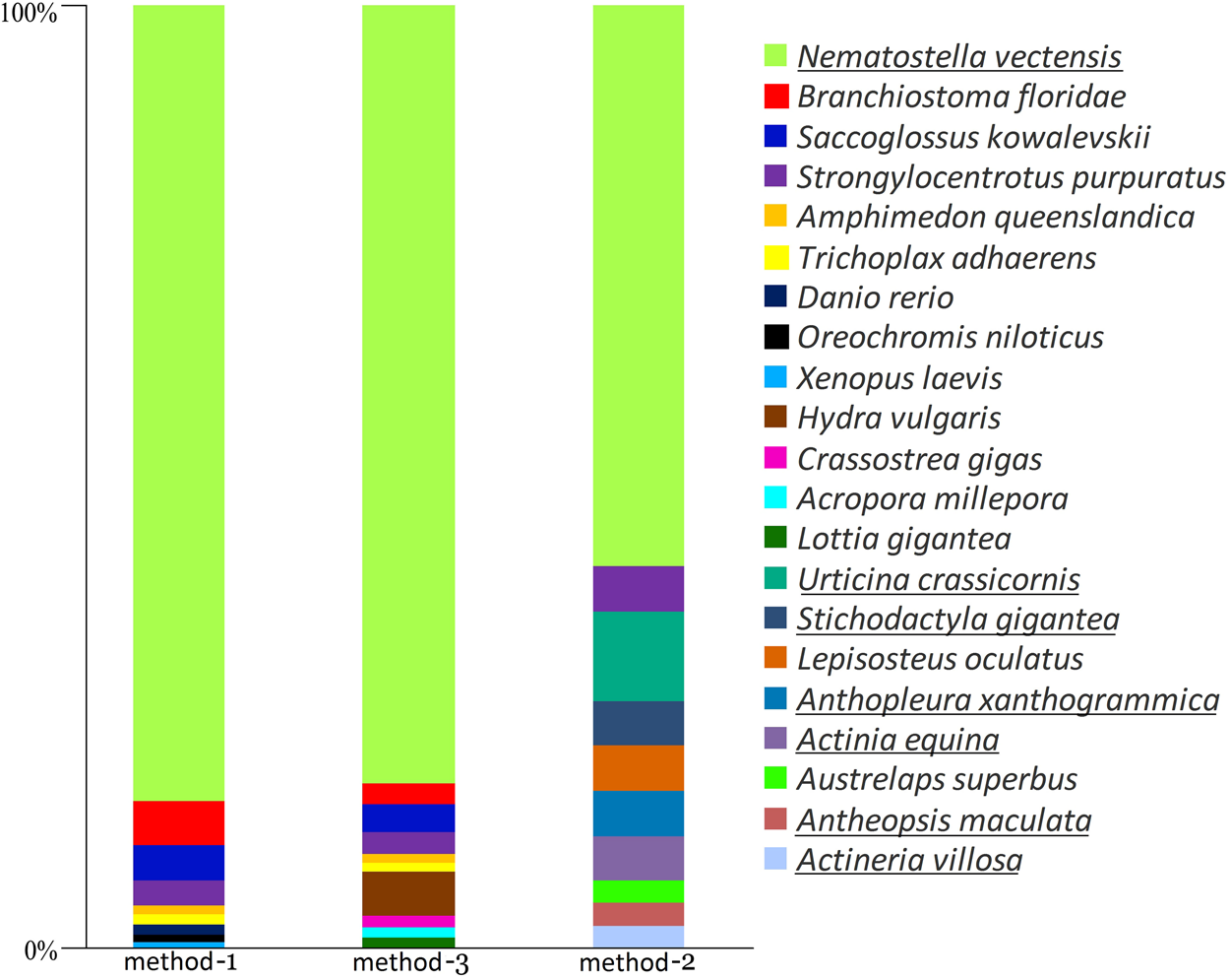
Top 10 BLAST hits for organisms with different assemblies of sample *C. japonicus* Yellow. Sea anemone species are underlined.

The results of the assemblies obtained using methods 1, 2, and 3 were subjected to further analysis and annotation. Figure 2 shows the data derived from a comparison of the consensus sequences with the BLAST NR sequence database for *C. japonicus* Yellow; the results for *C. japonicus* Red were similar. The criterion used for comparison was the taxonomic similarity of the sequences. As shown in the diagram, the most significant change in distribution between the species was revealed using method 2.

Regarding the transcript coverage of the different functions, method 1 produced the most diverse distribution, while the other 2 methods were more specialised. A detailed GO (gene ontology) analysis of the isotigs obtained using method 1 revealed different functional categories of detected components (Figure S1). Items representing cellular, single-organism, and metabolic processes were dominant in the biological processes category; items representing binding and catalytic activity were dominant in the molecular function category; and items representing cellular and organelle molecular functions were dominant in the cellular components category. The GO differences between the 2 specimens were not statistically significant.

Moreover, the transcripts obtained from method 1 were annotated using KOG classification. The qualitative distribution of transcripts corresponding to different functional categories is shown in Figure S2. In summary, the predominant group of isotigs was annotated into categories including translation, ribosomal structure, and biogenesis, in agreement with the results of the GO analysis.

### Data validation through proteomics

The samples harvested for venom analyses were highly diluted by sea water; therefore, we concentrated the samples through solid-phase extraction on reverse-phase sorbents. This technique is convenient for polypeptide components but is not appropriate for large, secreted proteins, due to the reverse-phase features of C_18_- (very large and/or hydrophobic proteins may be absorbed permanently). Thus, polypeptide compounds were our major focus, rather than proteins. For validation, we used a high-performance LC-MS-MS technique that allows the identification of peptides in a multicomponent mixture, without preliminary separation stages and with high sensitivity. A united reference base of tryptic peptides was created from «Predicted protein set 1» (*C. japonicus* Yellow: 4410 sequences; *C. japonicus* Red: 5149), «Predicted protein set 2» (*C. japonicus* Yellow: 354; *C. japonicus* Red: 358), and «Predicted protein set 3» (*C. japonicus* Yellow: 1208; *C. japonicus* Red: 1392); this reference base was then used to identify venom compounds according to their molecular weight and sequence tag.

For validation by proteomics, several criteria were applied. It was necessary for at least 2 peptides to align with each individual sequence in one of the predicted protein sets. The tryptic peptides of interest covered 16–73% of the particular sequences in the predicted protein sets. The distribution of the peptide fragments using B-Y sequence tags in fragmentation spectra was taken into account. Not all of the transcriptome-predicted components were validated by proteomics; in total, 27 polypeptide and protein sequences were validated in the *C. japonicus* Yellow and *C. japonicus* Red samples, including 5 large secreted protein molecules and 22 polypeptide molecules. Furthermore, we focused only on proteomics-validated molecules.

**Figure 3:**
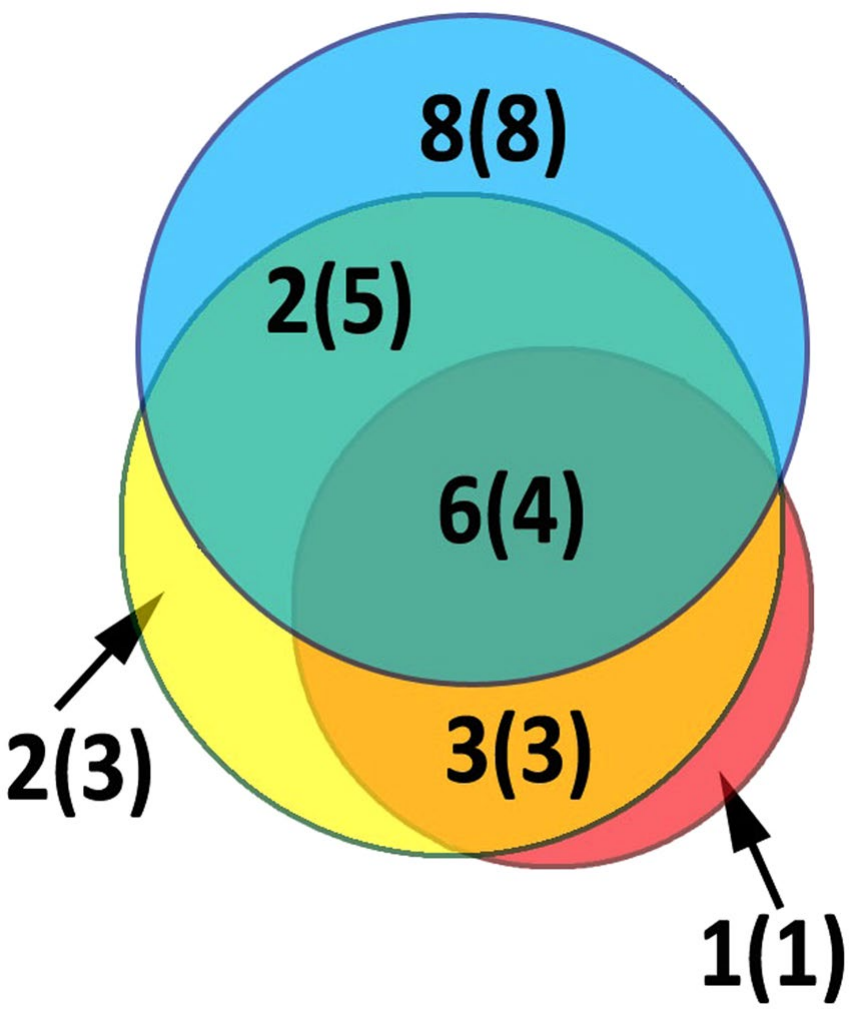
Venn diagram for validation of *C. japonicus* Yellow with values for *C. japonicus* Red in brackets. Data are coloured: red for method 1, yellow for method 2, and blue for method 3.

Each transcriptome data analysis method resulted in specific sets of polypeptides (Fig. 3). Method 1 resulted in the smallest number of validated toxins, while method 3 was the most sensitive. The validated toxins identified by all 3 methods were homologues of the classical components of sea anemone venoms APEKTx1, Kunitz-type chymotrypsin inhibitor, gigantoxin-II, gigantoxin-III, and BDS-I but exhibited moderate or low homology.

### Toxins of C. japonicus

On the basis of their structural homology with known molecules, the polypeptides that were validated using proteomics were subdivided into 3 groups: neurotoxic peptides, toxin-like molecules and *C. japonicus* polypeptides. According to the nomenclature proposed by Kozlov^28^, the neurotoxic peptides were designated Cj anemonotoxins (AnmTX Cj). Because the yellow and red specimens belonged to a single species and their venoms showed very similar compositions, no further subdivision according to method or specimen was necessary. The 3 employed methods resulted in 3 different sets of data, which exhibited substantial overlap. For example, the same 5 AnmTX Cj polypeptides showing the highest homology to UniProt toxins were identified and validated using each of the 3 methods.

**Figure 4:**
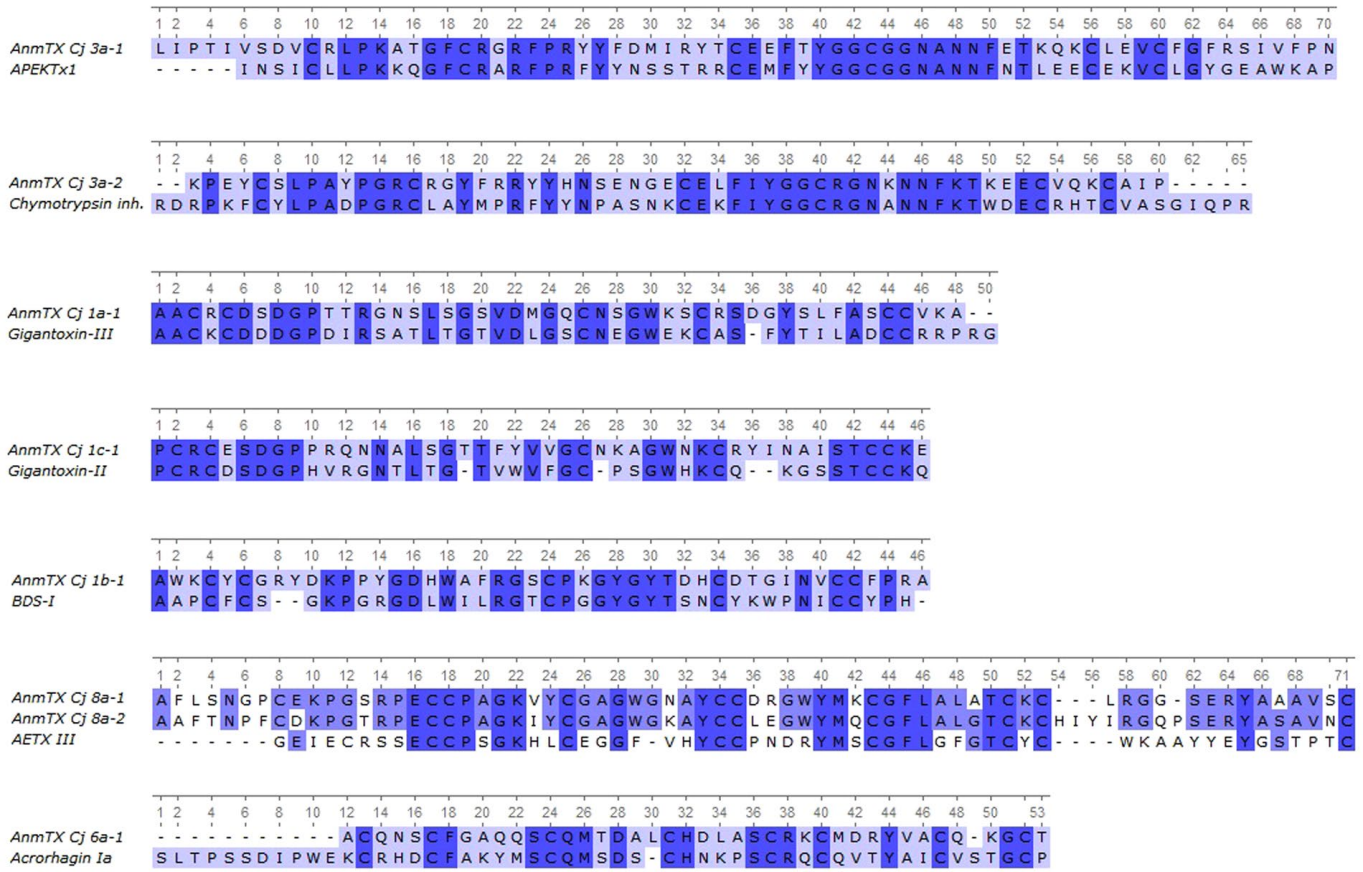
Alignment of mature toxin sequences from *C. japonicus* venoms with mature sequences of their homologues.

The AnmTX Cj group consists of homologues of sea anemone polypeptide toxins in the UniProt database; therefore, it is possible to make assumptions about the potential biological targets of these molecules. Primary structure similarity does not necessarily imply similar function; nevertheless, we considered it useful to mention the homology of the new molecules with toxins from UniProt. As noted above, according to primary structure characteristics (so-called Cys frameworks or Cys patterns), up to 16 groups of toxins can now be distinguished^28,29^. The components identified in *C. japonicus* venom cover a small portion of those 16 groups. Table 1 presents the sequences of mature toxins identified using this approach^30^. Fig. 4 shows the alignments of all of these toxins with their closest homologues, and the AnmTX Cj full precursor protein sequences are listed in Table S3.

**Table 1:** Neurotoxins of the *C. japonicus* sea anemone. Length (in number of aa residues) is shown for mature sequences.

Toxin	Length	Homolog	E-value	Identity	Organism	Cys-framework	Superfamily
AnmTX Cj 3a-1	70	APEKTx1	2e–15	46%	*Anthopleura elegantissima*	3a	Kunitz-type
AnmTX Cj 3a-2	59	Chymotrypsin inhibitor (P00992)	1e–14	55%	*Vipera ammodytes*	3a	Kunitz-type
AnmTX Cj 1a-1	48	Gigantoxin-III	6e–09	50%	*Stichodactyla gigantea*	1a	NaTX Type II
AnmTX Cj 1c-1	49	Gigantoxin-II	1e–05	52%	*S. gigantea*	new (1c)	NaTX Type I
AnmTX Cj 1b-1	46	BDS-I	4e–4	48%	*Anemonia sulcata*	1b	KTX type III
AnmTX Cj 8a-1	66	AETX III	2e–3	36%	*Anemonia erythraea*	8a	—
AnmTX Cj 8a-2	71	AETX III	0.027	37%	*A. erythraea*	8a	—
AnmTX Cj 6a-1	41	Acrorhagin Ia	0.880	29%	*Actinia equina*	6a	—

The E-value is taken from pBLAST results. The species from which the closest homologue was isolated, the Cys framework motif, and the superfamily in UniProt are shown. A light grey background marks the polypeptides that have a new cysteine distribution motif.

The identified toxins included AnmTX Cj 3a-1 and 3a-2, which are homologous to Kunitz-type peptides. This type predominantly consists of serine protease inhibitors with plesiotypic structural and functional toxin forms from various animals and K^+^ channel blockers from snake and sea snake venoms. Moreover, sea anemone toxins that modulate TRPV1 receptor activity were recently discovered to be in this structural class^31,32^. AnmTX Cj 3a-1 is homologous to the APEKTx1 toxin from the venom of the sea anemone *Anthopleura elegantissima*, which inhibits serine proteinases (for example, trypsin, with K_d = _124 nM) and selectively (and reversibly) inhibits the mammalian potassium channels rK_v_1.1 and KCNA1 (IC_50_ 0.9 nM)^33^. AnmTX Cj 3a-2 is homologous to a chymotrypsin inhibitor from the venom of the long-nosed viper *Vipera ammodytes* ^34,35^.

AnmTX Cj 1a-1 and AnmTX Cj 1c-1 are homologous to gigantoxin-3 and gigantoxin-2 from the sea anemone *Stichodactyla gigantea* ^36^. The targets for gigantoxin-3 and gigantoxin-2 have not yet been determined. Based on their homology to other, better understood toxins, it may be assumed that their function is to inhibit the inactivation of sodium channels (δ-toxins). Both toxins belong to the most abundant structural group of sea anemone toxins (group 1). Toxin AnmTX Cj 1b-1 also belongs to this group and shares similar residues with toxin BDS-I from *Anemonia sulcata* venom, whose biological activity has not yet been investigated^37,38^. Presumably, AnmTX Cj 1a-1, AnmTX Cj 1b-1, and AnmTX Cj 1c-1 are δ-type inhibitors of sodium channels. AnmTX Cj 8a-1 and AnmTX Cj 8a-2 are homologous to each other and to the AETX III toxin from the sea anemone *Anemonia erythraea*. The target of AETX III is not known, but it is toxic to crabs^39^. As their names indicate, both AnmTX Cj 8a-1 and AnmTX Cj 8a-2 belong to structural group 8a. This structural group has only a few members. Apart from the polypeptides presented in Fig. 4, only one toxin belongs to this group: AETX II, whose activity has not been studied in depth^39^. Therefore, it is difficult to speculate about the possible activities of AnmTX Cj 8a-1 and 8a-2.

AnmTX Cj 6a-1 belongs to structural group 6a and is homologous to acrorhagin-1a, which was isolated from the venom of the sea anemone *Actinia equina* ^40^. AnmTX Cj 6a-1 is the second representative of this structural group. Because nothing is known about acrorhagin-1a activity, no proposed activity of AnmTX Cj 6a-1 can be suggested at this time.

### Toxin-like molecules

**Figure 5:**
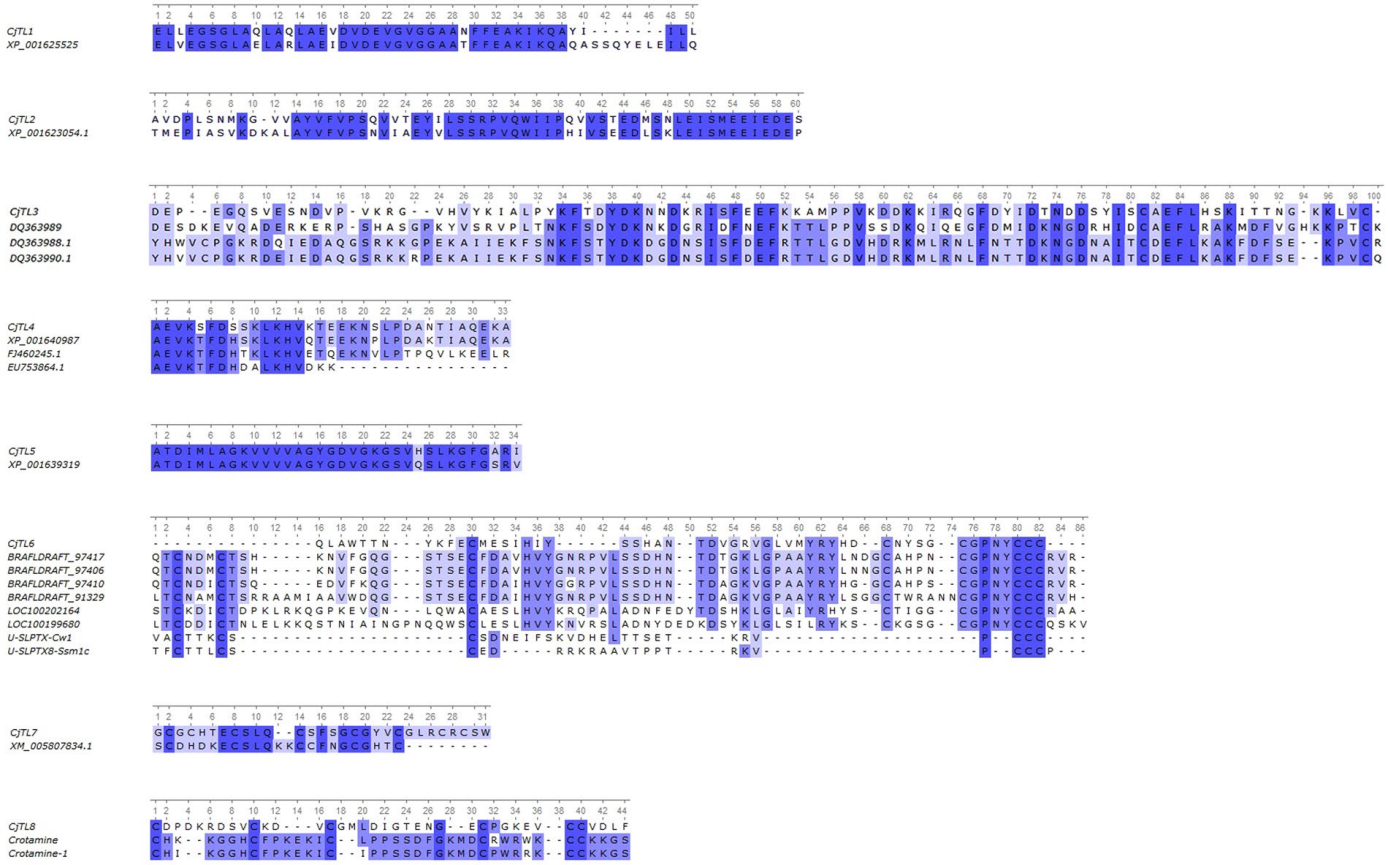
Alignment of the mature sequences of the toxin-like components of *C. japonicus*.

This group of *C. japonicus* toxin-like (CjTL) molecules consists of polypeptides that are homologous to predicted proteins. These predicted proteins were extracted from GenBank and cannot be found in the UniProt data bank, due to a lack of evidence regarding their existence at the protein level. Homology of the toxin-like molecules was observed not only with the full-length sequences of these proteins but also with partially resolved sequences (Fig. 5). All of the proteins homologous to CjTL polypeptides are hypothetical, or their functional annotation is predicted. In general, the mature sequences of the toxin-like components of *C. japonicus* exhibit lengths of less than 60 aa, similar to the lengths of other sea anemone toxins. The full sequences of the precursor proteins are listed in Table S3, and the homology parameters of the CjTL sequences are given in Table 2.

**Table 2:** Toxin-like components of the *C. japonicus* sea anemone.

Toxin-like	Length	Homolog seq ID	E-value	Identity	Organism	Cys-framework
CjTL 1	55	XP_001625525	8e–20	81%	*Nematostella vectensis*	linear
CjTL 2	62	XP_001623054	1e–17	66%	*N. vectensis*	linear
CjTL 3	93	DQ363989	3e–17	39%	*Aiptasia pallida*	10a
CjTL 4	52	XP_001640987	1e–16	85%	*N. vectensis*	linear
CjTL 5	34	XP_001639319	3e–11	91%	*N. vectensis*	linear
CjTL 6	52	BRAFLDRAFT_97417	1e–08	42%	*Branchiostoma floridae*	new
CjTL 7	30	XM_005807834.1	17	41%	*Xiphophorus maculatus*	new
CjTL 8	39	Crotamine (Q9PWF3)	1.3	22%	*Crotalus durissus terrificus*	new
CjTL9	36	No homology	—	—	—	linear

Length in aa is shown for mature sequences. The E-value is calculated as a result of implementing the pBLAST algorithm for closest homologues. Sequence IDs and species names are denoted for the items to which each toxin has its closest homology. Cys framework accessories are also shown. The light grey colour marks the CjTL sequences that have new motifs of cysteine distribution.

CjTLs 1, 2, 4, 5, and 9 are linear, cysteine-free polypeptides. Similar molecules have previously been identified in the venoms of sea anemones^24^ and shown to primarily possess cytolytic activity^41^. It is well established that venom cytolytic activity exists in the overwhelming majority of venomous marine animals, due to the presence of large linear molecules such as actinoporins and cytolysins^42–44^, rather than shorter polypeptides. However, smaller linear molecules with cytolytic and antimicrobial activities have been described in the venoms of many terrestrial animals^2,45,46^. Therefore, the length of the amino acid chain is not the defining factor for cytotoxic action. Both CjTLs and large actinoporins may possess this activity.

Amongst cysteine-containing sequences, it is important to note that CjTL3 shares Cys framework 10a. Moreover, in terms of domain types, CjTL3 may be relegated to the EF-hand superfamily. The EF-hand superfamily consists of proteins with a calcium-binding effect, which is peculiar to proteins such as troponin-C and calmodulins^47^. The EF-hand domain is characteristic of some snake proteins, including calglandulins and reticulocalbin-2. Although calglandulins are thought to be involved in the cell mechanism controlling the secretion of toxins from the glands into venom^48–50^, reticulocalbin-2 is found in the venom itself ^51^. Reticulocalbin-2 (alternative name: taipoxin-associated calcium-binding protein 49 homologue) is a 49 aa protein that was isolated from the venom of the snake *Crotalus adamanteus*, where its presence was further validated through transcriptomic^52^ and proteomic^53^ analyses. Therefore, in contrast to the calglandulins, reticulocalbin-2 is an authentic venom component in the EF-hand domain family; thus, this domain is not unknown to occur in venom protein components. Hence, CjTL3 may represent a novel, shorter member of the EF-hand superfamily. The calcium-binding function of EF-hand proteins may be used for diversification or to modulate venom action.

Three additional polypeptides (cysteine-rich) appeared to contribute to the composition of *C. japonicus* venom, and these polypeptides exhibit non-standard Cys frameworks. CjTL6 contains 3 vicinal cysteine residues at the *C*-terminus. This packing of cysteine residues is not a new feature among the predicted toxin sequences of sea anemones^54–56^. However, all of these examples are toxins containing 8 and 10 cysteine residues, while CjTL6 contains 6 cysteine residues, which therefore represent a new structural motif.

CjTL7 is homologous to a very small part of a predicted anosmin-1-like protein from the moonfish *Xiphophorus maculatus* (GenBank: XM_005807834.1). Anosmin-1 is a glycoprotein that is associated with an extracellular matrix that influences an organism’s development. The unprocessed anosmin-1 protein has a length of 543 aa and shares only 12 *N*-terminal aa residues with CjTL7 (see Fig. 5).

CjTL8 shows low homology to any protein (either predicted or from UniProt). The distribution of cysteine residues in CjTL8 is new for sea anemone toxins but is analogous to that observed in crotamine peptides, which have previously been found in rattlesnake venom and exhibit cell-penetrating and potassium channel-inhibiting properties^57^. Therefore, CjTL8 may share at least its similar spatial organisation with crotamines.

Despite its lack of structural homology, CjTL9 was included in the CjTL group because it contained a signal peptide and was derived by implementing method 3, which focused on research regarding sea anemone toxin sequences.

### C. japonicus polypeptides and venom proteins

In the group of *C. japonicus* polypeptides (CjPP), 5 precursor proteins were found (Table S3) to be quite long polypeptide molecules (60–130 aa). No close homologues were identified using pBLAST. These molecules are likely to be components of sea anemone venom, rather than representing false-positive results, because they passed through multiple filters.

In total, 21 large proteins were deduced for the 2 *C. japonicus* sea anemone specimens (Table S4), but only 5 of these proteins were validated by proteomics, including 2 sea anemone cytotoxic proteins, 2 zinc-dependent metalloproteinases, and a protein belonging to the MAC/Perforin domain family.

A protein designated CjVP1, belonging to the MAC/Perforin domain class, with a full length of 254 aa, was found to be partly homologous to Toxin AvTX-60A from the sea anemone *Actineria villosa* ^58^. AvTX-60A is lethal to mice and displays haemolytic action towards red blood cells^58^. Sea anemone cytotoxic proteins (cytolysins) are important and well-studied components of sea anemone venoms. Both *C. japonicus* cytolysins (CjVP2 and CjVP3) are homologous to urticinatoxin and bandaporin from sea anemones. Haemolytic activity has been reported for proteins of this group^1,24^.

Proteinases (CjVP4 and CjVP5) were verified to be present via mass spectrometry. These proteins belong to a group of zinc-dependent metalloproteinases and display the highest homology to аstacin-like metalloproteinase toxins 1/3/4, which are components of the venoms of the spiders *Loxosceles laeta* and *Loxosceles intermedia* ^59^. Some transcript-only zinc-dependent metalloproteinases share homology with nematocyst-expressed protein 6 from the sea anemone *Nematostella vectensis*. One set of transcripts also encode homologues of zinc-dependent metalloproteinase Nas4/9 from the nematode *Caenorhabditis elegans*.

As components of venoms, zinc-dependent metalloproteinases participate in the degradation of extracellular matrix proteins such as fibronectin, fibrinogen, and gelatin, thereby speeding up the distribution of venom in the prey’s tissues^60,61^. The determination of the functional roles of zinc-dependent metalloproteinases in venom is complicated, due to their high homology with proteinases that participate in cell processes such as proliferation, tissue remodelling, and apoptosis. To verify the presence of these proteinases, a phylogenetic tree was built using a neighbour-joining algorithm from PHYLIP with bootstrap analysis (see Figure S3). As a result, 5 proteinases were chosen as probable candidates for venom components (Table S4); however, only 2 of these proteinases were validated by proteomics.

### Expression and activity of the predicted toxins

Toxins and toxin-like molecules are important venom compounds, whose biological activity is oriented towards prey capture or defence. To test the toxicity of these molecules, we selected three candidates for production. Polypeptides with novel and unusual Cys-frameworks were chosen for expression.

We obtained the peptide AnmTX Сj 1c-1 (see Table 1) and 2 predicted toxin-like peptides, CjTL7 and CjTL8 (Table 2). CjTL7 and CjTL8 exhibit low structural homology to known sea anemone neurotoxins. Briefly, we expressed the target toxins in *E. coli* with the aid of vectors based on the pET32 plasmid. The expression of short peptides was assisted by fusion with the thioredoxin protein, and the target toxins were cleaved from the fusion product after reaction with cyanogen bromide (CNBr)^62^. Each fusion protein was designed with additional methionine residues at the *N*- and *C*- termini of the mature sequence to ensure cleavage success (see Figure S6). The CjTL8 toxin contains Met-16, which was replaced with Leu-16 to prevent toxin degradation (Table S6). As a result, recombinant analogues of the toxins were produced with an additional *C*-terminal methionine residue that was converted to homoserine or/and homoserine lactone by reaction with CNBr^63^. Polypeptide toxins (for use as controls) were designed and produced in a similar way. Other experimental details of recombinant peptide production are included in the supplementary materials and methods. The purity of the recombinant toxins and the presence of *C*-terminal homoserine or/and homoserine lactone were confirmed by mass spectrometry (Figures S7–S11, Table S6). Biological tests of the obtained peptides (which are described below) confirmed that the Met → Leu modification and the *C*-terminal modifications did not abolish the activities of the recombinant toxins.

To test the toxic activity of the peptides, we used aquatic and terrestrial animals: the shrimp^64^ *Caridina multidentata* and fly larva^65^ of *Musca domestica* ^66^, respectively. All of the recombinant sea anemone toxins produced neurotoxic effects on the shrimp, ranging from a slight twitching of the walking legs (pereiopods) to immediate and severe paralysis, followed by death. The effect of CjTL7 on the shrimp was weak, causing meandrous behaviour, representing a mild behavioural change in the shrimp (for more detailed description see Table S9). No lethal effect was observed in this case.

**Figure 6:**
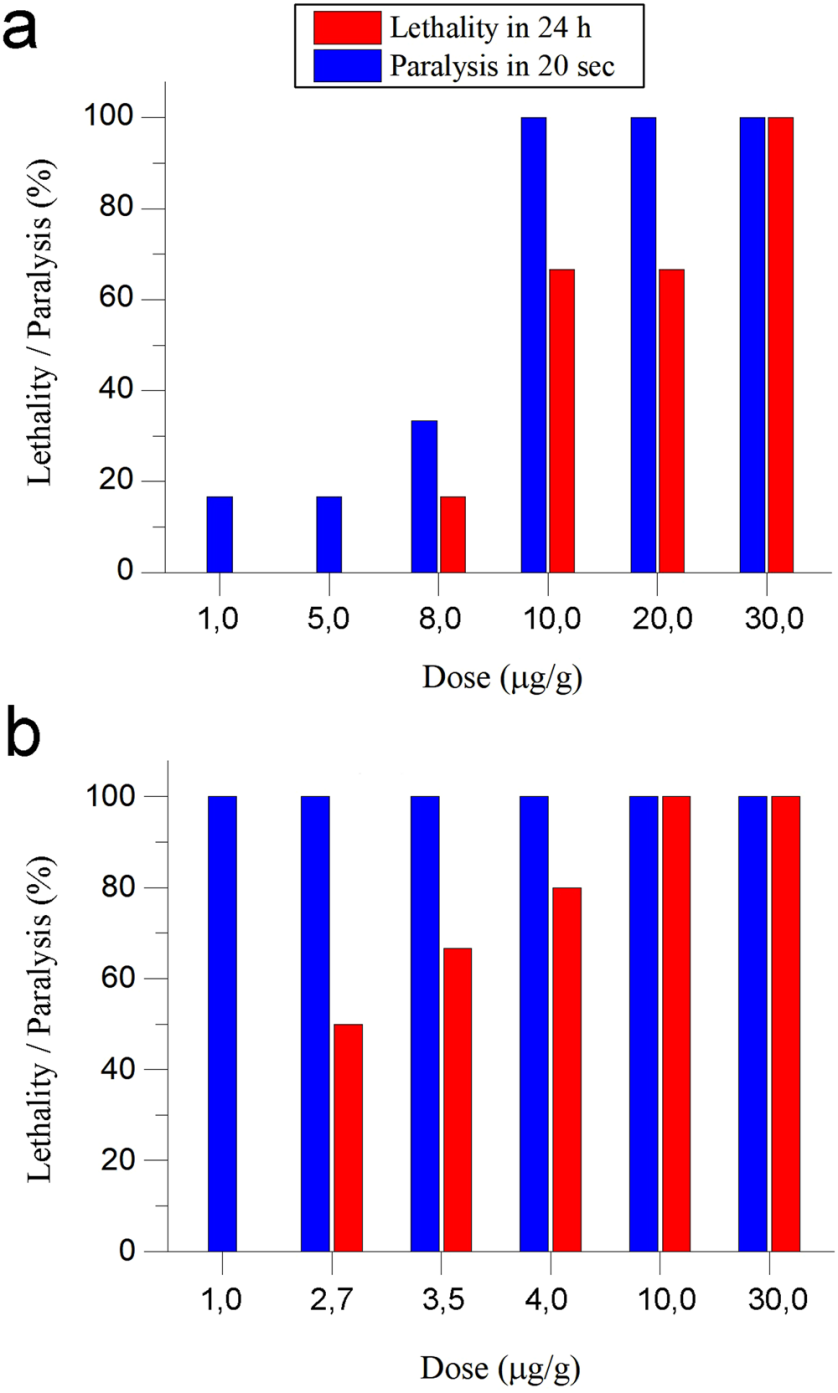
Lethal and paralysis effects of new toxins (**a**) AnmTx Cj 1c-1 (**b**) CjTL8 on crustaceans tested by intraperitoneally injection to *Caridina multidentata* shrimps. Various doses in the range of 1.0 ÷ 30.0 μg/g were tested. Effects were monitored during 24 h. Lethality (in 24 h) and immediate paralysis efficiencies are shown.

AnmTX Сj 1c-1 and CjTL8 exhibited far more severe activity. Injection of AnmTX Сj 1c-1 or CjTL8 induced marked paralysis of the shrimp at 10–20 sec after injection (see Videos S1 and S2). Among all of the tested toxins, CjTL8 was the most potent and induced immediate paralysis, regardless of the injected dose. When high doses of the toxins were applied, the shrimp showed no remission from paralysis over the period of 24 h. No movement other than convulsive tremors of the pereiopods and pleopods of the shrimp was observed during the first 40 min of paralysis. This effect was dose-dependent. In particular, the high dose of the toxins killed the shrimp (no further movements over 24 h). Lower doses were only partially lethal to the animals, and some shrimp exhibited full remission within 12 h (see Fig. 6, Table S7, Table S8). AnmTX Сj 1c-1 had an LD_100_ of 30 μg/g, while CjTL8 had an LD_100_ of 10 μg/g.

Two types of controls were used. Recombinant δ-actitoxin-Cgg1a (UniProt: P0C280), as described by Ständker *et al*.^67^, possessing a uniquely strong crab-paralyzing activity (LD_50_ of approx. 1 μg/kg according to Ständker *et al*.^67^) was used as a positive control. δ-Actitoxin-Cgg1a was highly lethal to the shrimp (see Video S3) following injection at a dose of 30 μg/g. A recombinant mutant of δ-actitoxin-Cgg1a in which all cysteine residues were substituted with serine residues was employed as a negative control. The same dose (30 μg/g) of mutated δ-actitoxin-Cgg1a produced no observable effect on the shrimp (see Video S4).

**Figure 7:**
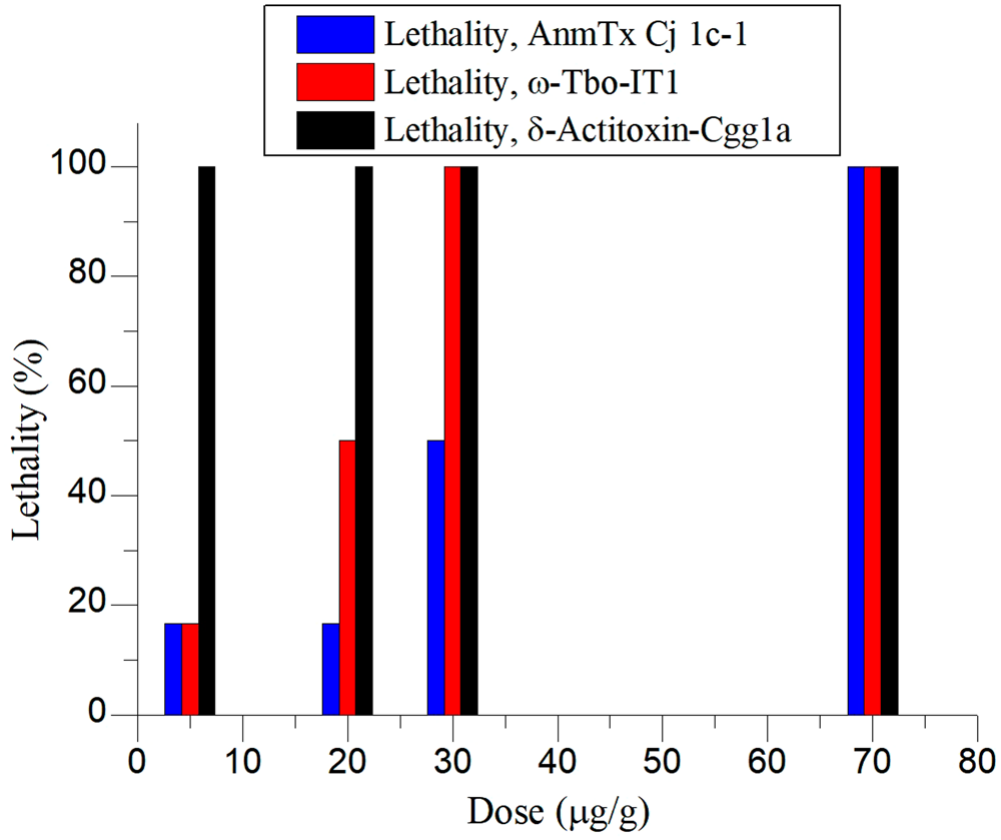
Activity of toxin AnmTx Cj 1c-1 on insect larvae *Musca domestica*. Lethality ratios are shown for various doses. AnmTx Cj 1c-1 – new toxin identified in this research work; ω-Tbo-IT1 – insecticidal spider toxins which serves as positive control; δ-actitoxin-Cgg1a – peptide possessing high toxicity on both crustaceans and insect, serves as second positive control.

The insect experiments showed that CjTL7 and CjTL8 lacked toxic effects on the fly larvae, while AnmTX Сj 1c-1 exhibited weak toxicity to the insects, with an LD_50_ of 30 μg/g (Fig. 7 and Table S10). The insecticidal spider toxin ω-Tbo-IT1 (ω-phylotoxin-To1a)^66^ and δ-actitoxin-Cgg1a^67^ were used as positive controls. Both toxins showed potent toxic activity towards the insects (LD_100_ of 5 μg/g for δ-actitoxin-Cgg1a and 30 μg/g for ω-Tbo-IT1, see Fig. 7). Moreover, two negative controls were used in the experiments with insect larvae: physiological saline buffer and the Cys → Ser mutant of δ-actitoxin-Cgg1a. Neither of the negative controls induced any paralysis or death.

## Discussion

The investigation of animal venom components using NGS sequencing technology is not an innovative idea. The standard, often-used method involves short-read assembly and annotation, combined with proteomic validation, which we also employed in this work. Such analyses have been carried out for various venomous animals, including spiders^68^, snakes^69^, scorpions^11^, cone snails^8,17^, bees^70^, centipedes^71^, jellyfish^72^, fish^10^, mosquitoes^73^, and sea anemones^12^.

This combined approach allows us not only to predict hypothetical proteins but also to validate their presence in venom and even (potentially) perform relative quantification—for example, using MS quantification approaches. However, our experience shows that the actual/predicted ratios for validated proteins are far from ideal. Additionally, earlier implementation of sequencing for long, unfragmented transcripts (EST sequencing) may allow better identification of the components^11,16,74^.

The most likely reason for this issue is that the assemblers fail to correctly assemble protein genes that are very similar in primary structure and short, such as animal toxins generated based on combinatorial principles (duplicated genes with high homology). As a result, transcripts that disregard single substitutions or consolidate several similar sequences into one frequently appear. Therefore, improved data processing protocols should be developed for toxin identification to improve success. We reduced the size of the initial raw-read dataset using the aforementioned approaches for excluding inconsistent raw reads.

A reference database containing all known toxins (the UniProt Animal Toxin Annotation Program) was used as a BlastX reference. This database contains more than 6,000 polypeptides and proteins from animal venoms that have been isolated not only from *Cnidaria*, but also from snakes and the Arthropoda and Mollusca phyla. Using this database, we separated definite groups of reads that corresponded to parts of animal toxins.

In method 3, the emphasis was placed mainly on the cysteine-rich polypeptide components of venom; therefore, the sea anemone toxins from UniProt (and the predicted toxins from TrEMBL) were subdivided into 16 groups using motifs related to the Cys distribution; the results became a reference database. We sorted the reads according to 16 structural groups after comparing the initial set of reads with each protein from each structural group. Therefore, the reads could be separated employing a single toxin reference sequence (as in poorly represented structural groups) and, for example, the 116 sequences in group 1a. Using this approach, the very large sets of initial data were sorted; only 1.8% of the sequences were selected for further analysis. Despite this decrease from the number of reads in the initial set, the results were well correlated with those obtained using methods 1 and 2. Moreover, among the 27 validated components, 8 were exclusively found using method 3.

Method 1 was quite successful for large venom protein identification: all of the 5 proteins identified by proteomics were deduced using method 1 (and one was only deduced using this method). This method was also effective in identifying toxins that exhibited high homology levels to known toxin polypeptides. However, unlike method 2, this approach cannot be used for the identification of neurotoxins with novel structures and cannot predict toxin-like sequences (as method 3 can). We consider method 1 a good approach for rapidly assessing the diversity of major venom components that resemble known toxins. For deeper investigation of venom component diversity, we recommend using a combination of all 3 methods.

The high specificity of the annotation for the top 10 organisms in the different read assemblies is notable. This is reflected in Fig. 2, which shows the results of analysis of the *C. japonicus* Yellow sample, where most transcripts are annotated similarly to transcripts of *N. vectensis*. This finding may be explained, first, by the similarities between these two organisms (*C. japonicus* and *N. vectensis*) and by the intensive research on the sea anemone *N. vectensis* (whose genome has been sequenced and annotated). However, the percentage of similarity with *N. vectensis* is different in all 3 cases. For example, standard transcripts assembled using method 1 exhibit 82% identity with *N. vectensis*. Interestingly, under method 3, in which the initial read database was substantially reduced in size, the deduced contigs show analogous identity to *N. vectensis*, and their resemblance to other organisms is also quite high. The initial read database was reduced by approximately 50-fold when using method 2 compared with method 3, causing the percentage of similarity with *N. vectensis* to decrease by up to 60%, which also caused the appearance of such organisms as *Urticina crassicornis* (sea anemone), *A. equina* (sea anemone), *Lepisosteus oculatus* (gar), *Anthopleura aff*. (sea anemone), *S. gigantea* (sea anemone), and *Strongylocentrotus purpuratus* (sea urchin). It is noteworthy that some of these newly appearing organisms do not produce venoms, such as the gar.

The discovery of toxins with novel folds is important in venomics because it is likely that some folds have not yet been discovered. We identified new motifs of the distribution of cysteines in *C. japonicus* toxins, which might indicate possible new folds. Hence, AnmTX Cj 1c-1, CjTL7, and CjTL8 were objects of exceptional interest in this study. We decided to experimentally determine whether the AnmTX Cj 1c-1, CjTL7, and CjTL8 peptides actually possess biological activity by testing their lethal and paralytic effects on shrimp and flies.

One novel motif was found for the AnmTX Cj 1c-1 toxin, which is a particular variation of the classical motifs 1a and 1b. Most likely, there is a more general framework for the toxins of sea anemones: C1C##C[6–9]C-[6–9]-CC#. If so, we can expect the future discovery of polypeptides with a gap of 8 aa between residues Cys-III and Cys-IV. AnmTX Cj 1c-1 exhibited both paralytic and lethal effects on the shrimp *C. multidentata* and was lethal to the insects at a high dose.

Among the identified toxin-like molecules, CjTL6 should be mentioned. This molecule contains 3 vicinal cysteine residues in the *C*-terminus. Overall, this motif may be represented as C27C4C4-CCC. Jouiaei *et al*.^54^ recognised a similar, but not identical, motif including 3 vicinal cysteine residues using BLAST in their research on polypeptide toxins based on sequencing data from *Anemonia viridis* and *Metridium senile*. Earlier, similar molecules referred to as SCRiPs (secreted cysteine-rich proteins) were described only as reef-building coral genes that are down-regulated by heat stress^55^. Recently, Logashina *et al*. reported^56^ a new sea anemone toxin, τ-AnmTx Ueq. 12–1 (short name, Ueq. 12–1), which has 10 cysteine residues with 3 vicinal cysteine residues in the *C*-terminus. This toxin exhibits antimicrobial activity and potentiates the TRPA1 receptor^56^. Therefore, C-terminal motifs containing 3 vicinal cysteine residues are not a novel discovery among sea anemone toxins. However, CjTL6 is the first of these toxins identified that possesses only 6 cysteine residues to the best of our knowledge.

Another candidate for harbouring a novel sea anemone toxin fold is CjTL7, containing a non-standard symmetrical cysteine distribution motif, С1С-3-С3С-4-С3С-3-С1С3, which was previously unknown among natural polypeptides. CjTL7 is a rather short toxin comprising only 29 amino acid residues, with 8 cysteine residues. In our toxicity assays, recombinant CjTL7 affected the shrimp in a more complex manner than AnmTX Cj 1c-1: CjTL7 induced alternating periods of paralysis and strong convulsions but was not lethal.

There are two main 8-cysteine frameworks of sea anemone toxins: 6a and 11b (see Table S2). Each of these frameworks is represented by just one toxin of approximately 50 aa in length; framework 6a is represented by acrorhagin-I, and 11b is represented by MsePTx1. Acrorhagin-I is a 50-residue polypeptide and is toxic to crabs (LD_50_ 520 µg/kg)^40^. MsePTx1 (GenBank FC839755.1) is a hypothetical toxin derived from a cDNA sequence obtained from the tentacles of the sea anemone *Metridium senile*. Eight-cysteine toxins are typically found in cone snail venoms. However, the Cys framework exhibited by CjTL7 is not found in cone snail toxins.

Another intriguing compound, CjTL8, exhibited the strongest paralytic and lethal effects on the shrimp *C. multidentata* and resembles some snake venom proteins, due to its cysteine residue framework: C8C-3-C11C-5-CC6. Such a framework is common for many snake toxins—for example, Crotamine-1 from the pit viper *Crotalus durissus terrificus*, also known as myotoxin-1^57^. Crotamine-1 has not been studied in detail; however, based on its homology to other well-investigated myotoxins, it can be assumed to exhibit cell-penetrating and K_v_-inhibiting activity. The homology of CjTL8 to crotamine-1 is poor. These molecules are identical only in terms of their Cys framework, but we assume that this might indicate potential neurotoxic activity of CjTL8.

It may be assumed that CjTL7 possesses some modulatory function and plays a supporting role in the action of the venom, while AnmTX Cj 1c-1 and CjTL8 are responsible for the main lethal effect. The biological role of other components of *C. japonicus* venom was not explored in our work and warrants future investigation.

## Conclusion

In this report, we describe the use of transcriptomics and proteomics methods for the compositional analysis of the venom of the cold-water sea anemone *C. japonicus*. The use of a combination of 3 methods to search for toxins and toxin-like sequences yielded more structures than classical approaches. The venom compositions of the 2 specimens differed insignificantly, and we assume that analysing larger transcriptome datasets or increasing the number of biological replicates would show that the venoms of these anemones are identical, which is not a self-evident result.

As our data suggest, *C. japonicus* venom contains disulphide-rich toxins, linear polypeptides, and large venom proteins; among the toxin molecules, it is worth mentioning the homologues of Kunitz-type protease inhibitors and voltage-gated ion channel inhibitors, which are usual components of sea anemone venom. The venom proteins of *C. japonicus* include cytolytic proteins, zinc-dependent metalloproteinases, and phospholipases. However, low-homology sequences were also identified at the mRNA and protein levels, and some of these sequences exhibited new structural motifs (new Cys frameworks).

To confirm the biological significance of the molecules exhibiting new structural motifs, we expressed 3 of the unusual toxins in *E. coli*. The biological activity of the new toxins (AnmTX Cj 1c-1, CjTL7 and CjTL8), according to our experiments, shows preferential toxicity to crustaceans. CjTL8 exhibits the highest crustacean toxicity and selectivity among these new polypeptides.

## Materials and Methods

### Object sample

Two *C. japonicus* specimens (with yellow- and red-coloured bodies) were collected in the vicinity of Petropavlovsk-Kamchatsky using SCUBA equipment. The living specimens were kept in a cold-water aquarium for several months. To collect venom, specimens were placed on dishes with normal saline. After that, venom exudation was stimulated by electric impulses. Electrostimulation for venom release was conducted using a handmade stimulator (current parameters: 6.5 V and 10 Hz). The specimens were then placed back into the aquarium and were fed intensely. The venom-collection procedure was repeated several times, in 3-week intervals.

### cDNA library preparation and sequencing

*Tentacle treatment*. Tentacles were cut off using scissors, frozen in liquid nitrogen, and stored at –70 °C up to RNA isolation. Total RNA was extracted with TRIzol Reagent (Life Technologies, US). The yield and purity were assessed using a Nanodrop ND-1000 spectrophotometer (Thermo Scientific, US), with the RNA integrity determined by the RNA integrity number using a Bioanalyser 2100 (Agilent Technologies, US). The PCR-based cDNA library was created following the instructions for the Mint-2 cDNA synthesis kit (Evrogen, Russia). Adapters PlugOligo-3M and CDS-4M were used for cDNA synthesis.

*Ion torrent sequencing*. For library preparation, amplified cDNAs (100 ng of each sample) were fragmented by 400–500 bp using the Covaris S220 System (Covaris, Woburn, Massachusetts, US). Next, the Ion Xpress Plus Fragment Library Kit (Life Technologies) was employed for barcode shotgun-library sample preparation. To conduct emulsion PCR, the Ion PGM Template OT2 400 Kit (Life Technologies) was utilised. Sequencing was performed in accordance with manufacturer protocol for the genome analyser Ion Torrent PGM (Life Technologies), using an Ion 318 chip and Ion PGM Sequencing 400 Kit v2 (Life Technologies).

### Venom preparation

Immediately after stimulation, venom was washed from the animal with 10 mM EDTA and 1 mM PMSF solution. The obtained extract was concentrated on a solid-phase extraction Sep-Pak Vac C18 6 cc cartridge (Waters, US) using a 70% solution of acetonitrile (by volume) containing 0.1% TFA as a buffer for protein fraction elution. The acetonitrile was evaporated, and the remaining solution was lyophilised. The dry peptide powder was stored at –70 °C prior to mass spectrometric analysis.

### Venom sample digestion

A portion of 20 µg of lyophilised venom was resuspended in 20 µL of solvent containing 100 mM of NH_4_HCO_3_, 10 mM of DTT (Bio-Rad, US), and 0.1% RapiGest (Waters), after which the resulting mixture was heated to 100 °С for 5 minutes. It was further subjected to centrifugation at 14 000 rpm for 5 minutes. The supernatant was collected, and protein concentration was determined using a Bradford Protein Assay Kit (Bio-Rad). Iodoacetamide solution in 100 mM NH_4_HCO_3_ was added to achieve a final concentration of 3 mM. The mixture was incubated for 30 minutes, in the dark, at room temperature. DTT was then added to a final concentration of 10 mM and incubated for 20 minutes at ambient temperature. The sample, after being reduced and alkylated, was digested by trypsin (Trypsin Gold, Mass Spectrometry Grade; Promega, US) at a 1:50 trypsin to protein ratio for 16 hours at 37 °С without changing the buffer solution. The digestion reaction was stopped with the addition of 10% TFA solution with a pH value between 2 and 3. After 45 minutes, precipitate formed, and it was then separated by centrifugation (at 14 000 rpm for 10 minutes); the supernatant was then desalted on С18 SPE Discovery DSC-18 cartridges (50 mg and 1 mL; Supelco, US), which were preliminarily washed with 1 mL of methanol and 1 mL 0.1% TFA solution. Samples were washed with 1 mL of 5% acetonitrile in 0.1% TFA and were further eluted by 1 mL of 50% acetonitrile solution in 0.1% TFA. Elution fractions were concentrated to 2 µL and diluted in 3% acetonitrile solution in 0.1% formic acid to reach a volume of 10 mL.

### Proteomics

Analysis was performed on a TripleTOF 5600 + mass spectrometer with a NanoSpray III ion source (ABSciex, US) coupled with a NanoLC Ultra 2D + nano-HPLC system (Eksigent, US). The HPLC system was configured in a trap-elute mode. For the sample loading buffer and buffer A, the mixture of 98.9% water, 1% methanol, and 0.1% formic acid (by volume) was used. Buffer B was 99.9% acetonitrile and 0.1% formic acid (by volume). Samples were loaded on a Chrom XP C18 trap column (3 µm, 120 A, and 350 µm × 0.5 mm; Eksigent) at a flow rate of 3.5 µL/min for 10 minutes and eluted through a 3C18-CL-120 separation column (3 µm, 120 A, and 75 µm × 150 mm; Eksigent) at a flow rate of 300 nL/min at 35 °C. The gradient was from 5% to 40% of buffer B for 90 minutes followed by 10 minutes at 95% of buffer B and a 20-minute equilibration at 5% of buffer B. The blank 90-minute run of a wave form (5%-95%-5% of buffer B) was applied between samples to wash the system and to prevent carryover. β-Galactosidase tryptic solution (20 fmol) was run after every 2 samples using a 15-minute gradient (5–25% of buffer B) to calibrate the mass spectrometer and to control the system’s overall performance, stability, and reproducibility.

### MS data acquisition and analysis

A fully automated method was utilised. An information-dependent mass spectrometer experiment included 1 survey scan in MS1 followed by 50 dependent MS2 scans. The MS1 acquisition parameters were as follows: positive mode, mass range 300–1250 m/z, and signal accumulation time 250 milliseconds. Ions for MS2 analysis were selected based on an intensity above the threshold of 200 cps and a charge state from 2 to 5. MS2 acquisition parameters were as follows: resolution of quadrupole set to UNIT (0.7 Da), measurement mass range of 200–1800 m/z, and signal accumulation time of 50 milliseconds for each parent ion (using a 50-millisecond signal accumulation time). Analysed parent ions were sent to the dynamic exclusion list for 15 seconds to get an MS2 spectra at the chromatographic apex. Raw LC-MS/MS data were used for peptide identification with ProteinPilot (version 4.5) software (ABSciex) using the following parameters: Cys alkylation by iodoacetamide, trypsin digestion, and thorough ID search with detected protein threshold of 95.0% against the database.

### Shrimp-toxicity tests

This type of toxicity was measured on adult *Caridina multidentata* freshwater shrimps. These shrimps may be divided into 2 groups based on their size and weight: medium and large. Mean weights were estimated using an analytic balance. The medium shrimps weighed about 0.250 g, and the large ones weighed about 0.450 g. Recombinant toxin samples were lyophilized and dissolved in MQ water. Serial dilutions (by volume) were used to obtain sets of solutions with different concentrations of toxins. These solutions were used to produce different doses of toxins. Exact sets of doses were lined up after the preliminary toxicity test. Each dilution was made by taking into account that the volume of injection should ideally be no more than 5 µL and no less than 1 µL. Injections were made using a 10 μL syringe (Hamilton, US). Toxins samples were injected intraperitoneally into the shrimp. Fatal toxicity was observed 24 h after injection. Generally, for each dose of toxin, 6 shrimps were injected.

Two types of negative controls were employed. The first was a physiological solution of 150 mM sodium chloride, which was injected in parallel with and in the same number of shrimps as the investigated toxin. The second type of negative control included 2 high doses of bovine serum albumin to test whether high doses of sporadic protein molecule would have any effect on shrimps. Changes in the shrimps’ behaviour were monitored thoughtfully for 24 hours after the injections, and all behaviour deviations from the negative controls were recorded in detail according to the protocol. The following doses of AnmTX Cj 1c-1 were tested: 1.0, 5.0, 8.0, 10.0, 20.0 and 30 mg/kg. The following doses of CjTL7 were tested: 10.0, 25.0, 50.0, 100.0, and 150.0 mg/kg. Finally, the following doses of CjTL8 were tested: 1.0, 2.7, 3.5, 4.0, 10.0 and 30 mg/kg.

### Insect-toxicity tests

This type of toxicity was measured on *Musca domestica* larvae. The protocol we used was based on canonical often-used insect-toxicity protocol described by Eitan *et al*.^65^. The mean weight of larvae was experimentally estimated as 50 mg using analytic balance. Recombinant toxins were lyophilized and dissolved in MQ water. Fixed aliquots of resulting solutions were injected into larvae bodies with the help of 10 μL syringe (Hamilton, USA). To explore the dependence of effect from dose, for each toxin two different doses were tested. The first dose corresponded to lowest dose in shrimp-toxicity test; the second one corresponded to highest dose in shrimp-toxicity test. At least 18 larvae for each dose were subjected to injection. Changes in larvae behaviour had been overseen in the first 5 minutes after injection and hereupon after 24 h left. All larvae which were alive and not paralyzed after these 24 h were considered not affected by toxins. The same consideration was described in the original protocol^65^.

## Electronic supplementary material

Supplementary Video S1Injections and the action of AnmTX Сj 1c-1 toxin on shrimp

Supplementary Video S2

Supplementary Video S3

Supplementary Video S4

Supplementary Information
