# Supplementary material for: Identification of unusual peptides with new Cys frameworks in the venom of the cold-water sea anemone Cnidopus japonicus
Source: Sci Rep. 2017 Nov 6;7:14534. doi: 10.1038/s41598-017-14961-1 (PMC5673964; doi:10.1038/s41598-017-14961-1)
Supplement: Supplementary file 5 — Supplementary Information [file 41598_2017_14961_MOESM5_ESM.pdf]

Supplementary Information

Identification of unusual peptides with new Cys frameworks in the venom of the cold-water sea anemone *Cnidopus japonicus*

Vladislav V. Babenko, Alexander N. Mikov, Valentin A. Manuvera, Nickolay A. Anikanov, Sergey I. Kovalchuk, Yaroslav A. Andreev, Yulia A. Logashina, Daniil A. Kornilov, Alexander I. Manolov, Nadya P. Sanamyan, Karen E. Sanamyan, Elena S. Kostryukova, Sergey A. Kozlov, Eugene V. Grishin, Vadim M. Govorun, Vassili N. Lazarev

Supplementary Figure legends:

Fig. S1: Gene ontology by categories of biological process, cellular component and molecular function.

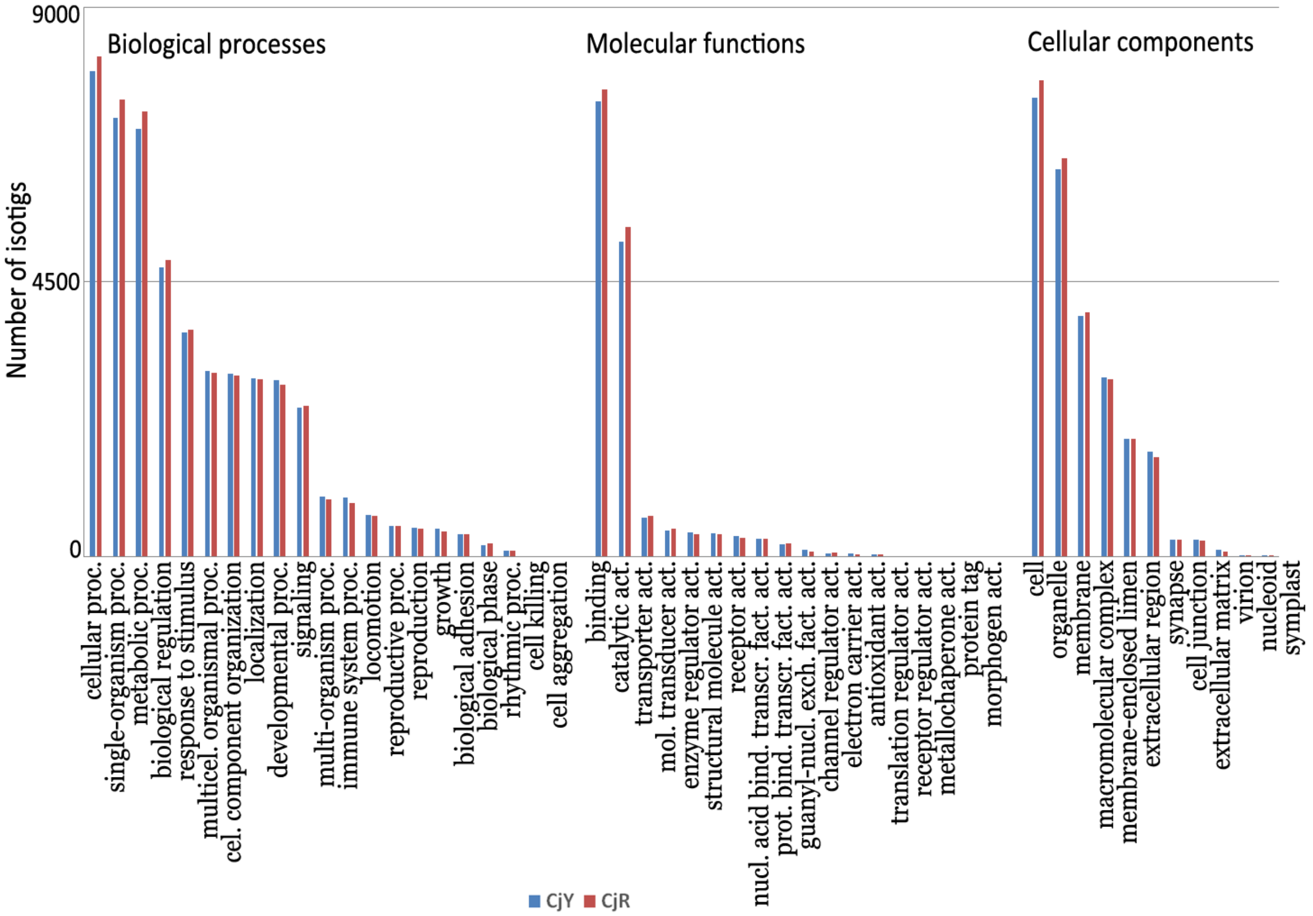

Fig. S2: Functional categories of the isotigs in KOG database.

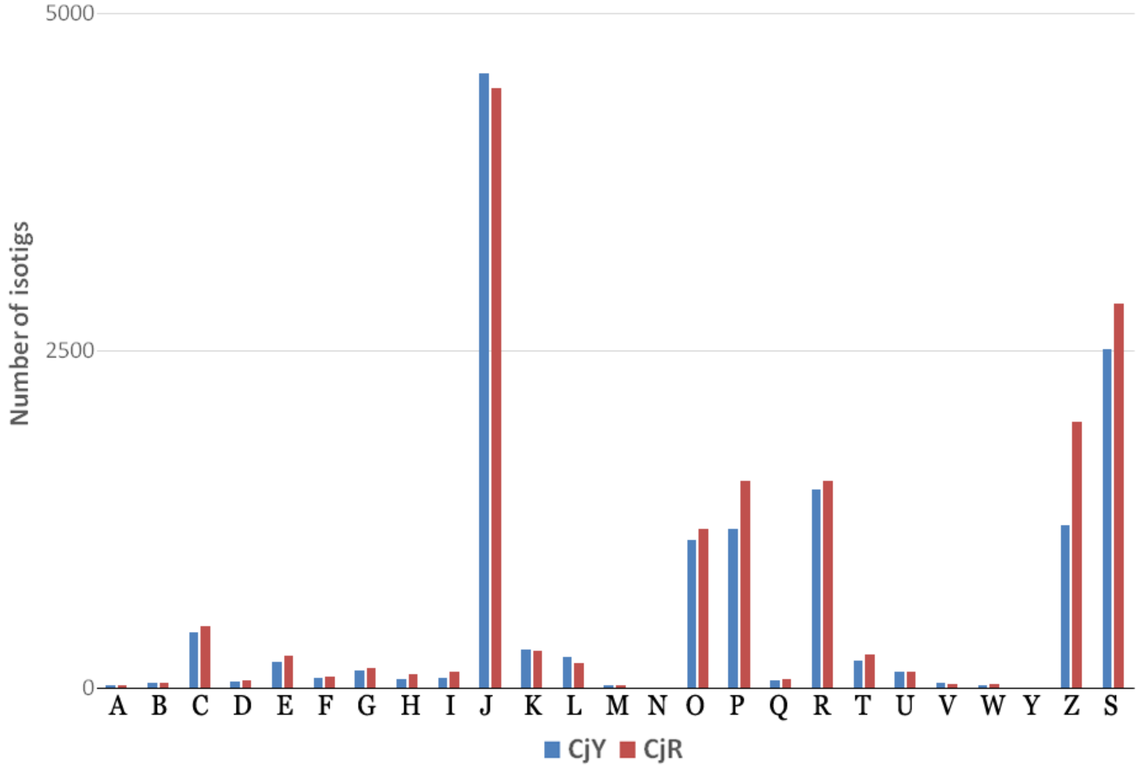

A, RNA processing and modification; B, chromatin structure and dynamics; C, energy production and conversion D, cell cycle control, cell division, chromosome partitioning; E, amino acid transport and metabolism; F, nucleotide transport and metabolism; G, carbohydrate transport and metabolism; H, coenzyme transport and metabolism; I, lipid transport and metabolism; J, translation, ribosomal structure and biogenesis; K, transcription; L, replication, recombination and repair; M, cell wall/membrane/envelope biogenesis; N, cell motility; O, posttranslational modification, protein turnover, chaperones; P, inorganic ion transport and metabolism; Q, secondary metabolites biosynthesis, transport and catabolism; R, general function prediction only; T, signal transduction mechanisms; U, intracellular trafficking, secretion, and vesicular transport; V, defense mechanisms; W, extracellular structures; Y, nuclear structure; Z, cytoskeleton; S, function unknown.

Fig. S3: Phylogenetic analysis of Zn-dependent metalloproteinases from *C. japonicus* transcripts.

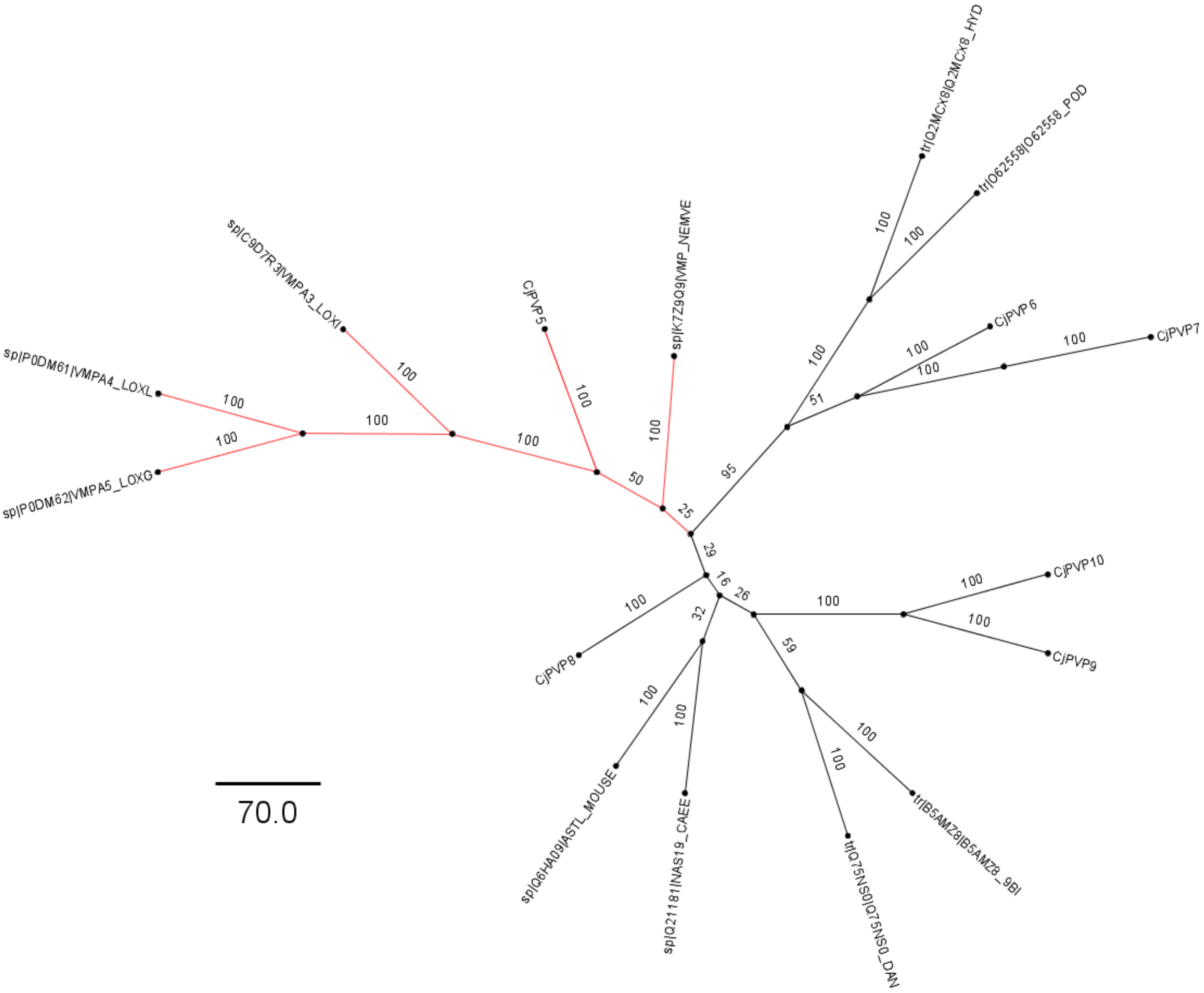

Unrooted radial tree of animal Astacin-like zinc metalloproteinases. Red branch combines zinc metalloproteases that represent known proteins of the venom and intrinsically are its active components. Therefore CjPVP5 presumably active component of venom. Parameters: *tree building method* PHYLIP Neighbor Joining, *distance matrix model* Jones-Taylor-Thornton, *bootstrapping and consensus tree* Majority Rule (extended). Key: CjPVP (5-10) – possible venom protein zinc-dependent metalloprotease from *C. japonicus*.

Accordance accession numbers:

sp|C9D7R3|VMPA3_LOXI - Astacin-like metalloprotease toxin 3, *Loxosceles intermedia* (brown spider)
sp|K7Z9Q9|VMP_NEMVE - Nematocyst expressed protein 6, *Nematostella vectensis* (starlet sea anemone)
sp|P0DM61|VMPA4_LOXL - Astacin-like metalloprotease toxin 4, *Loxosceles laeta* (brown spider)
sp|P0DM62|VMPA5_LOXG - Astacin-like metalloprotease toxin 5, *Loxosceles gaucho* (brown spider)
sp|Q21181|NAS19_CAEE - Zinc metalloproteinase nas-19, *Caenorhabditis elegans* (nematode)
sp|Q6HA09|ASTL_MOUSE - Astacin-like metalloendopeptidase, *Mus musculus* (house mouse)
tr|B5AMZ8|B5AMZ8_9BI - Astacin-like metalloendopeptidase *Ancylostoma ceylanicum* (nematode)
tr|O62558|O62558_POD - Astacin-like metalloendopeptidase, *Podocoryna carnea* (jellyfish)
tr|Q2MCX8|Q2MCX8_HYD - Astacin-like metalloendopeptidase, *Hydractinia echinata* (Snail fur)
tr|Q75NS0|Q75NS0_DAN - Astacin-like metalloendopeptidase, *Danio rerio* (zebrafish) (*Brachydanio rerio*)

Fig. S4: Example of pET32-anem1C-1 plasmid map. A) full map, B) fusion gene

A

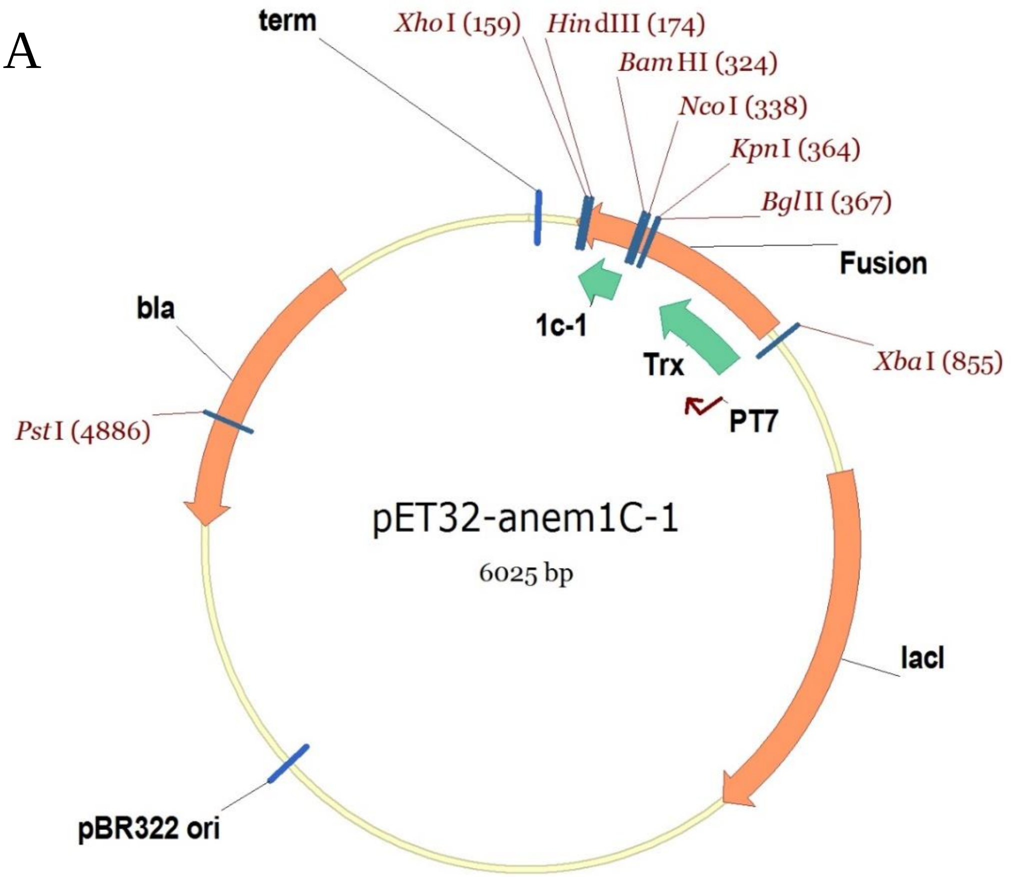

Trx - bacterial thioredoxin, **bla** - β-lactamase, **lacI**- Lactose operon repressor, **PT7** - T7 promotor, **term**-T7 terminator, HindIII(174) and BamHI(324) - restriction sites for DNA fragments, encoding toxins cloning.

B

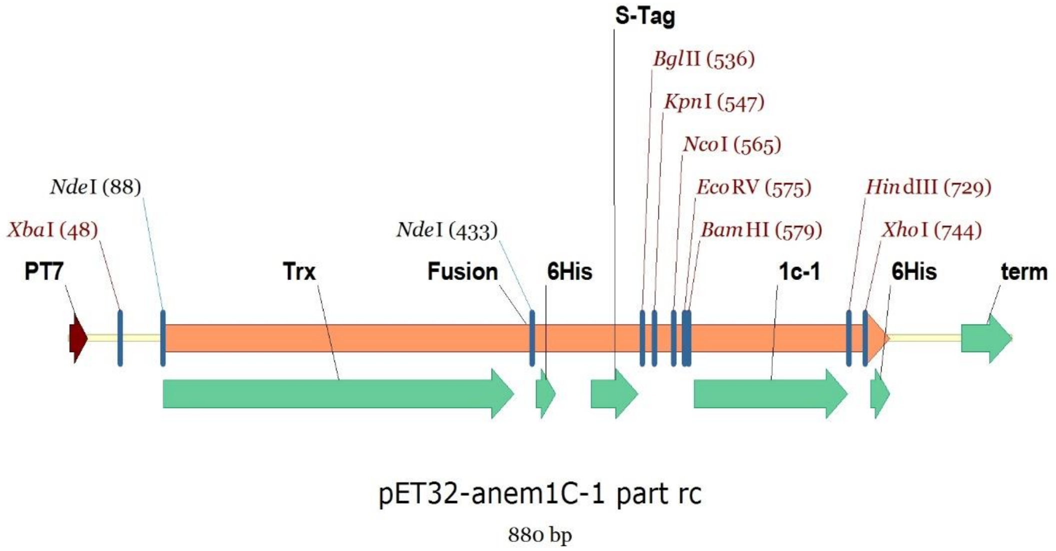

Trx - bacterial thioredoxin, **PT7** - T7 promotor, **term** -T7 terminator, **6His** - hexa histidine-tag, HindIII(729) and BamHI(579) - restriction sites for DNA fragments, encoding toxins cloning.

Fig. S5: SDS-PAGE gel electrophoresis scan. TrxA-Toxin fusion proteins were applied on the gel as well as standard SDS-PAGE markers. Short names of tracks imply: M – standard markers; 1c1 – fusion of AnmTX Cj 1c-1; TL7 – fusion of CjTL7; TL8 – fusion of CjTL8; X1 and X2 – two fusion proteins not used in this particular research; Cg – fusion of δ-actitoxin-Cgg1a; CgM – fusion of Cys→Ser mutant of δ-actitoxin-Cgg1a.

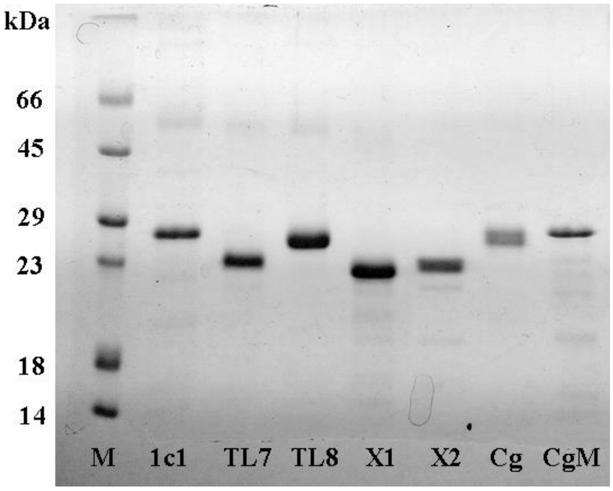

Fig. S6: Sequences of TrxA-Toxin fusion proteins (average *Mw*). N- and C- termini methionine residues (green), peptide sequence (yellow).

>TRX- ANMTX CJ 1C-1 (24541.78 Da)
MSDKIIHLTDDSFDTDVLKADGAILVDFWAEWCGPCKMIAPILDEIADEYQGKLTVAKLNIDQNPG
TAPKYGIRGIPTLLLFKNGEVAATKVGALSKGQLKEFLDANLAGSGSGHMHHHHHHSSGLVPRGS
GMKETAAAKFERQHMDSPDLGTDDDDKAMADIGSMPCRCESDGPPRQNNALSGTTFYVVGCNKA
GWNKCRYINAISTCCKEMKLAAALEHHHHHH
>TRX-CJTL7 (22591.63 Da)
MSDKIIHLTDDSFDTDVLKADGAILVDFWAEWCGPCKMIAPILDEIADEYQGKLTVAKLNIDQNPG
TAPKYGIRGIPTLLLFKNGEVAATKVGALSKGQLKEFLDANLAGSGSGHMHHHHHHSSGLVPRGS
GMKETAAAKFERQHMDSPDLGTDDDDKAMADIGSMGCGCHTECSLQCSFSGCGYVCGLRCRCS
WMKLAAALEHHHHHH
>TRX-CJTL8 (23461.56 Da)
MSDKIIHLTDDSFDTDVLKADGAILVDFWAEWCGPCKMIAPILDEIADEYQGKLTVAKLNIDQNPG
TAPKYGIRGIPTLLLFKNGEVAATKVGALSKGQLKEFLDANLAGSGSGHMHHHHHHSSGLVPRGS
GMKETAAAKFERQHMDSPDLGTDDDDKAMADIGSMCDPDKRDSVCKDVCGLLDIGTENGECPGK
EVCCVDLFMKLAAALEHHHHHH
>TRX- native δ-actitoxin-Cgg1a (24521.57 Da)
MSDKIIHLTDDSFDTDVLKADGAILVDFWAEWCGPCKMIAPILDEIADEYQGKLTVAKLNIDQNPG
TAPKYGIRGIPTLLLFKNGEVAATKVGALSKGQLKEFLDANLAGSGSGHMHHHHHHSSGLVPRGS
GMKETAAAKFERQHMDSPDLGTDDDDKAMADIGSMGVPCRCDSDGPSVHGNTLSGTVWVGSCA
SGWHKCNDEYNIAYECCKEMKLAAALEHHHHHH
>TRX- mutant δ-actitoxin-Cgg1a (24425.21 Da)
MSDKIIHLTDDSFDTDVLKADGAILVDFWAEWCGPCKMIAPILDEIADEYQGKLTVAKLNIDQNPG
TAPKYGIRGIPTLLLFKNGEVAATKVGALSKGQLKEFLDANLAGSGSGHMHHHHHHSSGLVPRGS
GMKETAAAKFERQHMDSPDLGTDDDDKAMADIGSMGVPSRSDSDGPSVHGNTLSGTVWVGSSAS
GWHKSNDEYNIAYESSKEMKLAAALEHHHHHH

Fig. S7: MALDI TOF mass spectrum with zoom for recombinant toxin δ-actitoxin-Cgg1a in the m/z range 500–6000. The peaks at 5024.4 and 2511.8 represent the single and double protonation states of the mutant of δ-actitoxin-Cgg1a peptide, respectively. In zoom view arrows showing C-terminal homoserine lactone and homoserine form.

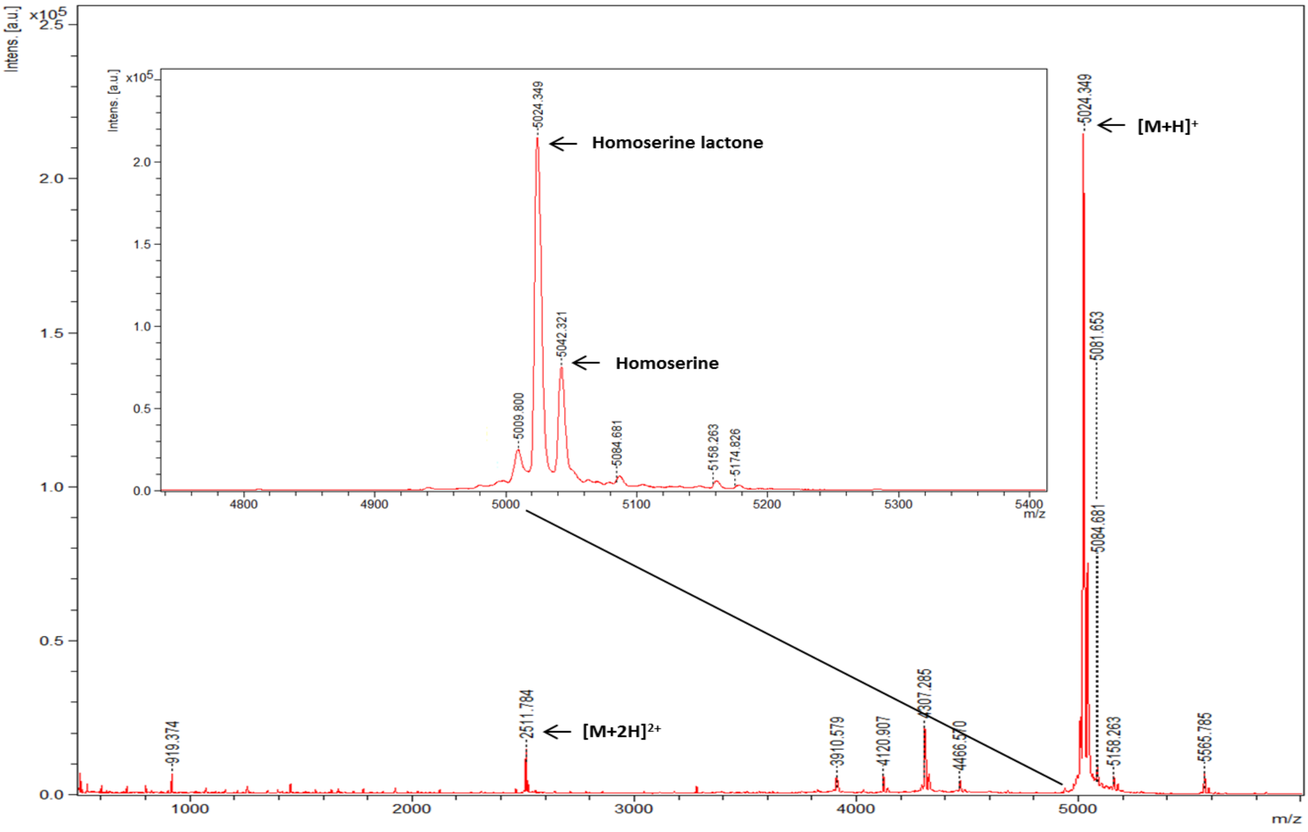

Fig. S8: MALDI TOF mass spectrum with zoom for Cys→Ser mutant of δ-actitoxin-Cgg1a in the m/z range 500–6000. The peaks at 5113.7 and 2556.5 represent the single and double protonation states of the native δ-actitoxin-Cgg1a peptide, respectively. In zoom view arrows showing C-terminal homoserine lactone and homoserine form.

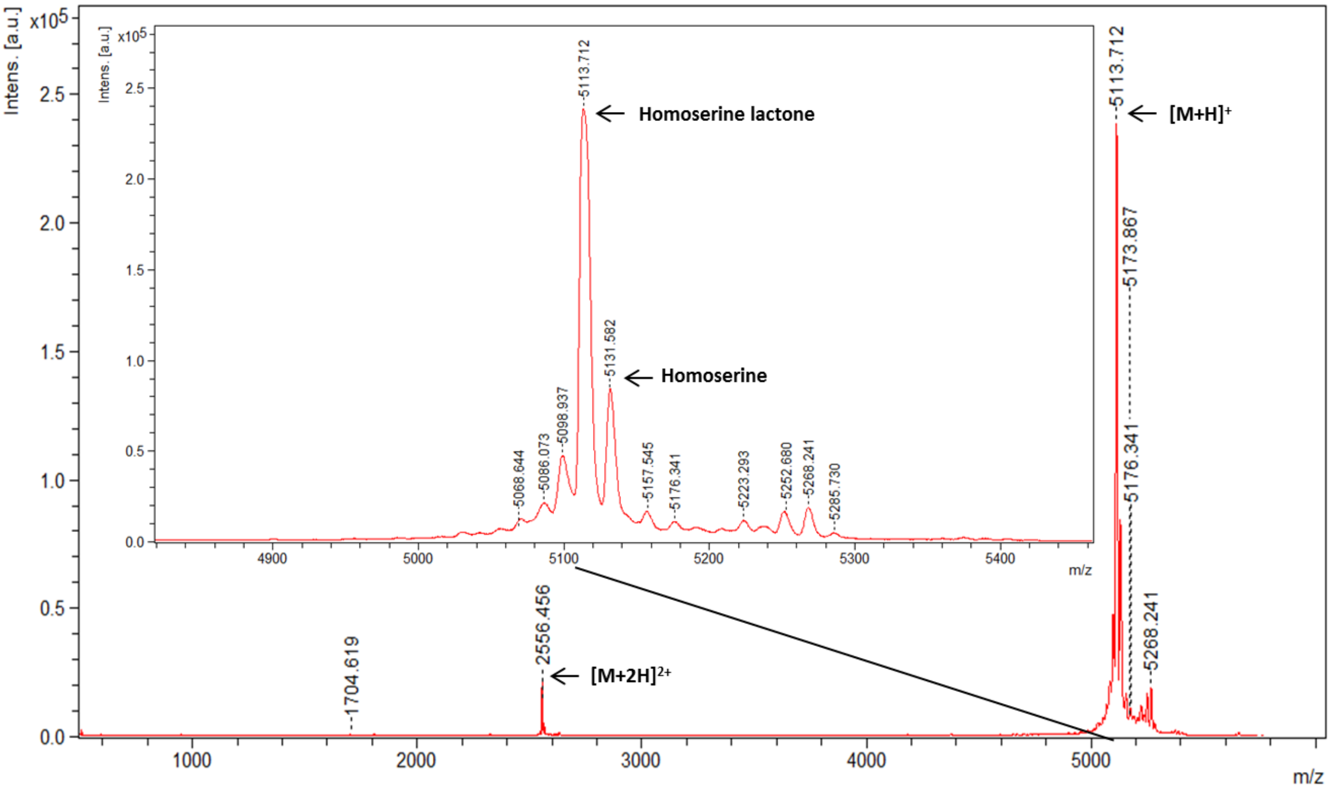

Fig. S9: MALDI TOF mass spectrum with zoom for AnmTX Cj 1c-1 in the m/z range 500–6000. The peaks at 5134.1 and 2567.4 represent the single and double protonation states of the AnmTX Cj 1c-1 peptide, respectively. In zoom view arrows showing C-terminal homoserine lactone and homoserine form.

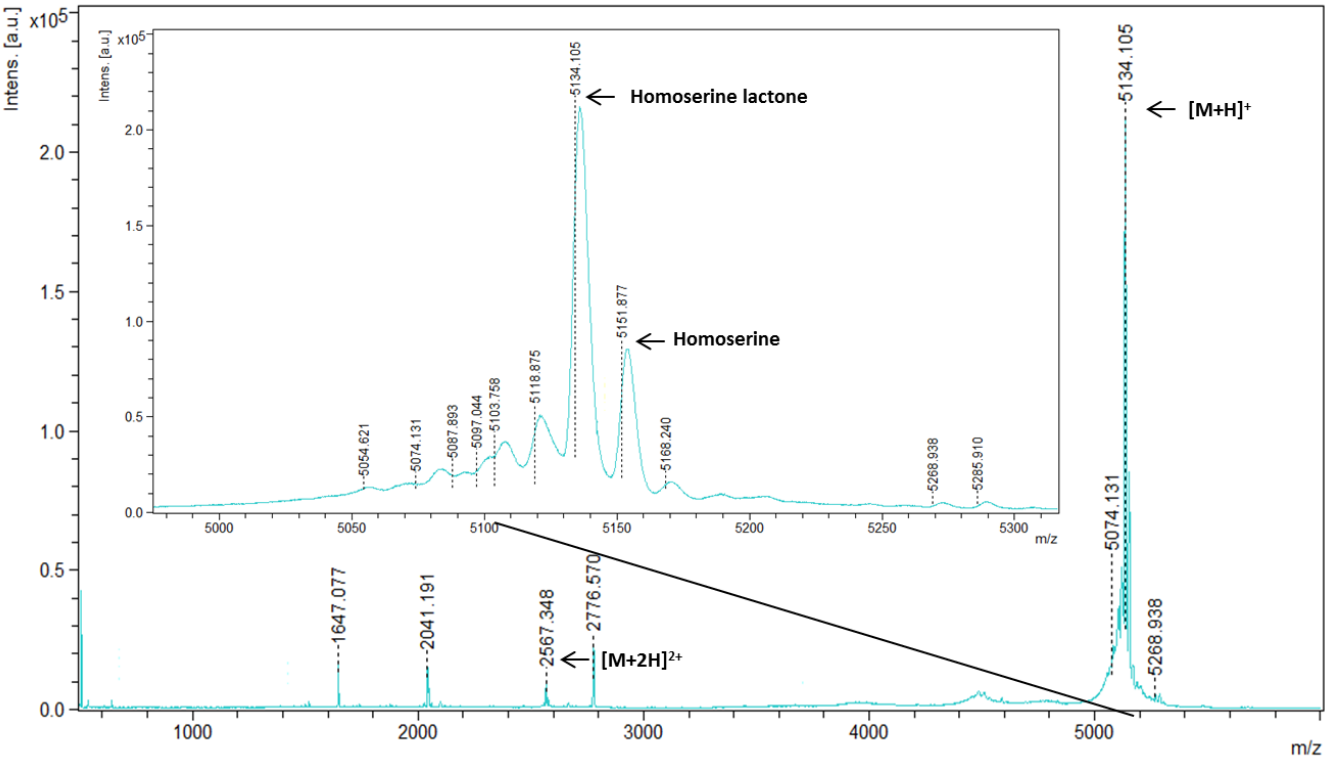

Fig. S10: MALDI TOF mass spectrum with zoom for CjTL8 in the m/z range 500–6000. The peaks at 4052.15 and 2027.5 represent the single and double protonation states of the CjTL8 peptide, respectively. In zoom view arrows showing C-terminal homoserine lactone and homoserine form.

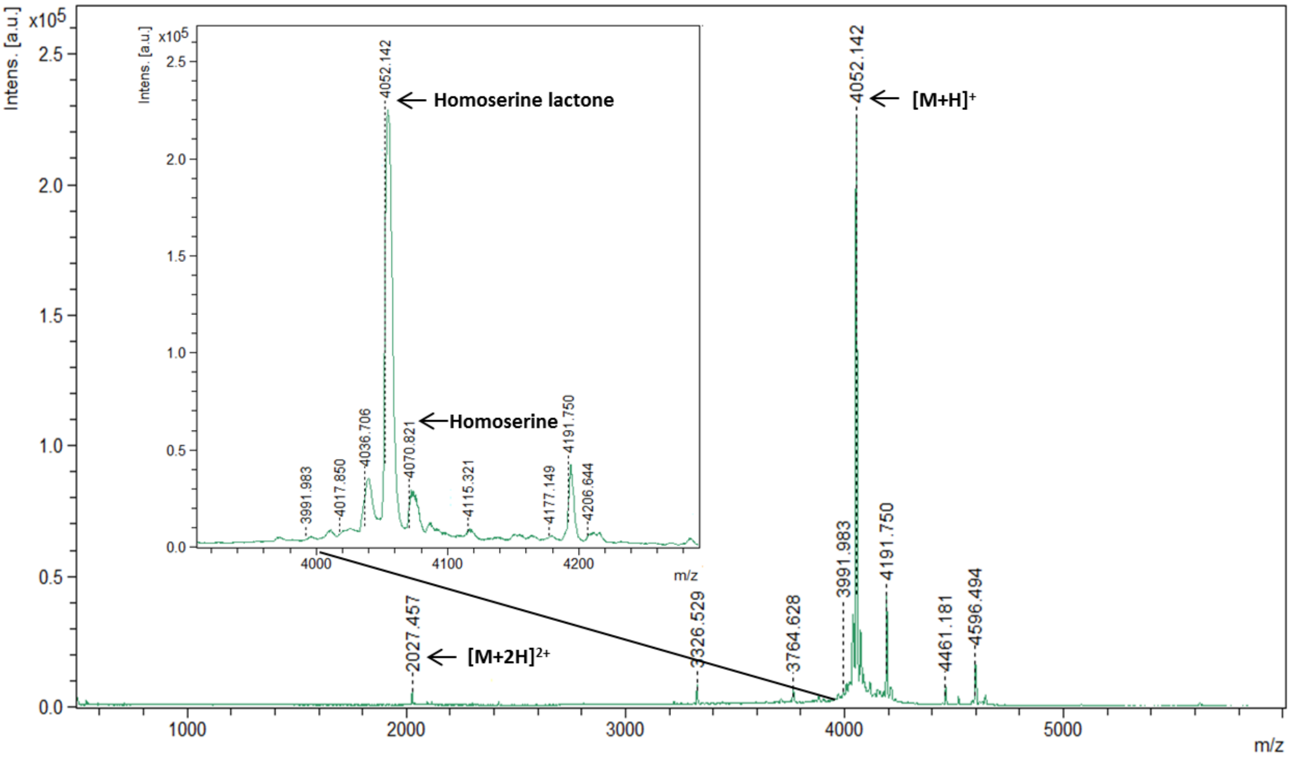

Fig. S11: ESI-Q mass spectrum with zoom for CjTL7 in the m/z range 200–2000. The peaks at 1067.8 the triple protonation states of the CjTL7 peptide. In zoom view arrows showing C-terminal homoserine and homoserine 1Cys-Cys reduced form.

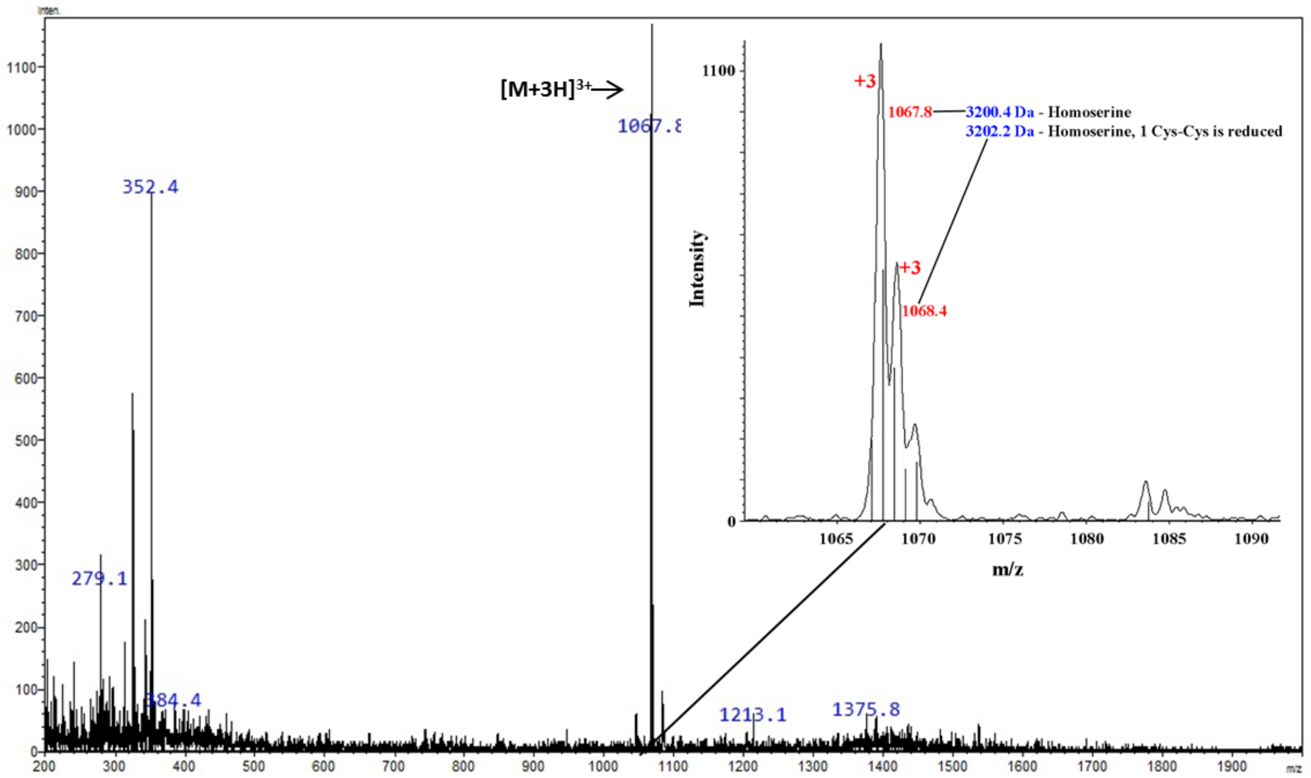

Supplementary tables:

Table S1. Statistical data for two sea anemone samples.

Sample	CjY	CjR
SRA id	**SRX1124372**	**SRX1124373**
Number of Bases	498,427,821	562,886,805
Q20 (quality score)	406,389,845	465,780,808
Reads	2,329,563	2,574,269
Mean Read Length	213 bp	218 bp
assembled by Newbler		
Number of Bases	30,979,384	30,991,069
Isotigs	45285	43599
Average size	684 bp	710 bp
Largest size	9097 bp	8332 bp

Table S2: Reference sequences used for sea anemone toxins search. Only UniProt sequences used as references are listed, although gene-deduced sequences were also used in the research.

Group №	Cys-frameworks	UniProt SEQ ID of sequence	Detected AnmTXs
1a	C1C##C6C-[6-9]-CC#	P01530, P19651, P0C280, P0C1F5, P0C1F4, Q7M425, P0C7Q0, P0C7P9, P82803, P01531, D2KX92, D2KX90, D2KX91, Q76CA3, A7SCE5, E3P6S4, B1B5I9, P01528, P01529, P01533, P01534, P08380, P0C1F0, P0C1F1, P0C1F2, P0C1F3, P0C5F4, P0C5F5, P0C5F6, P0C5F7, P0C5F8, P0C5F9, P0C5G0, P0C5G1, P0C5G2, P0C5G3, P0C5G5, P0C5G6, P0CH42, P10453, P10454, P30783, P30784, P30785, P30831, P30832, P69943, P86459, P86460, Q9NJQ2	AnmTX Cj 1a-1
1b	C1C##C9C-[6-8]-CC#	P61541, P61542, P11494, P59084, B3EWF9, P14531, P49127, P69930, P84919	AnmTX Cj 1b-1
2a	C8C-[4,8]-C*C3C2C	P29186, P29187, C0HJC2, C0HJC3, P81897, Q9TWG1	
2b	C6C#C*C3C#C	P11495	
2d	C3C#C*C6C##C	P16895	
3a	C8C15C7C12C3C	P31713, P81129, B1B5I8, C0HJF3, C0HJF4, P10280, P16344, P81547, P81548, A0A034WEL3, A0A095B2B5, A0A034WLZ5, P86862, B2G331	AnmTX Cj 3a-1, AnmTX Cj 3a-2
4a	C8C5C10C1C8C#	Q76CA1	
5a	CC1C-[2,4,5]-C5C4C#	P01535, P09949	
6a	C3C*C#C#C2C#C#C#	Q3C258	AnmTX Cj 6a-1
7a	C6C#C*C6C#C#	Q3C256	

8a	C#CC#C*CC#C#C1C*C	P69944, P69945	AnmTX Cj 8a-1, AnmTX Cj 8a-2
9a	C2C#C#C#	2LZO, P0C7W7, P69929	
10a	C##C#	P86465	CjTL3
11a	C6C#CC*C*C	C0HJB1, C0HJC4	
11b	C6C#CC*C*C*C1C#	P0DMD7	
Cys-free	–	Q5R231, P39088, Q8IAE2	CjPP1, CjPP2, CjPP3, CjPP4, CjPP5, CjTL1, CjTL2, CjTL4, CjTL5, CjTL9

Table S3: Sequences of protein precursors of toxins, toxin-like molecules and polypeptides of *C. japonicus*. Mature sequences are deduced using algorithm and highlighted by grey,[28] signal sequences are underlined, *pro-peptides* are shown in *Italics*.

Name	Precursor sequence	Prec. Length	Toxin Length
AnmTX Cj 3a-1	MARTANMAMVFLLSLVLVACVSLTNGLIPTIVSDVCRLPKATGFCRGRFPRYYFDMIRYTCEEFTYGGCGGNANNFETKQKCLEVCFGFRSIVFPN	96	70
AnmTX Cj 3a-2	MANKMVFLLCLLLVAGMAMAKPEYCSLPAYPGRCRGYFRRYYHNSENGECELFIYGGCRGNKNNFKTKEECVQKCAIPK	79	56
AnmTX Cj 1a-1	MASLKIVLIALMLLYTVSADIEVEKRAACRCDSDGPTTRGNSLSGSVDMGQCNSGWKSCRSDGYSLFASCCVKA	74	48
AnmTX Cj 1c-1§	MLNKRGVPCRCESDGPPRQNNALSGTTFYVVGCNKAGWNKCRYINAISTCCKEG	54	46
AnmTX Cj 1b-1	MAGSSITVFLGLMILCVVMVMASGEFREETVPMKRAWKCYCGRYDKPPYGDHWAFRGSCPKGYGYTDHCDTGINVCCFPRA	81	46
AnmTX Cj 8a-1	MIAKKVLCVFMLVVVLQQCFGMPDGFPAELRKRAFLSNGPCEKPGSRPECCPAGKVYCGAGWGNAYCCDRGWYMKCGFLALATCKCLRGGSERYAAAVSC	67	61
AnmTX Cj 8a-2	MIAKKVLCVFMLVVVLQQCFGMPDGFPAELRKRAAFTNPFCDKPGTRPECCPAGKIYCGAGWGKAYCCLEGWYMQCGFLALGTCKCHIYIRGQPSERYASAVNC	104	65
AnmTX Cj 6a-1	MHQLFSVLLVLGLMVYTVEALTAKKACQNSCFGAQQSCQMTDALCHDLASCRKCMDRYVACQKGCTRKRSYIPGAVMDLGRRLRLGD	87	62
CjTL1	MIKEVDEDNDGRVSFREFLLIFRKAAAGELLEGSGLAQLAQLAEVDVDEVGVGGAANFFEAKIKQAYIILLK	72	43
CjTL2	MYIPNTAVKISGEVIVSAKEFIFAFTNAHDVKDLPAAILNLSKKAVDPLSNMKGVVAYVFVPSQVVTEYILSSRPVQWIIPQVVSTEDMSNLEISMEEIEDESRRL	106	59
CjTL3*	MMIKVLLLLSSALVLFTPEAEGGCGCHTECSLQCSFSGCGYVCGLRCRCSWGKRDEPEGQSVESNDVPVKRGVHVYKIALPYKFTDYDKNNDKRISFEEFKKAMPPVKDDKKIRQGFDYIDTNDDSYISCAEFLHSKITTNGKKLVC	147	93
CjTL4	MPAELPDRSEVASFDQSKLKHVQTTEKNVIPNKDDIKTEAIETRAEVKSFDSSKLKHVKTEEKNSLPDANTIAQEKAKS	79	33

CjTL5	MKEKGTLKIPAINVNDSVTKSKFDNLYGIRESLVDGLKRATDIMLAGKVVVVAGYGDVGKGSVHSLKGFGARI	73	34
CjTL6	MAMSVCASLPSTLPGYIFPIRRACTSKSKETCAQVCKSAGVKKQLAWTTNYKFECMESIHIYSSHANTDVGRVGLVMYRYHDCNYSGCGPNYCCCRKGPQLKCVSA	106	52
CjTL7[*]	MMIKVLLLLSSALVLFTPEAEGGCGCHTECSLQCSFSGCGYVCGLRCRCSWGKRDEPEGQSVESNDVPVKRGVHVYKIALPYKFTDYDKNNDKRISFEEFKKAMPPVKDDKKIRQGFDYIDTNDDSYISCAEFLHSKITTNGKKLVC	147	29
CjTL8	MSSAIKILALLMVLVALAQA*KPRKDYRAYPDFDDKSVILEDDKR*CDPDKRDSVCKDVCGMLDIGTENGECPGKEVCCVDLFGR	83	37
CjTL9	MREHVMTEIDKDKDGFVSSDEFLSASKGDEFEKDEGWKGLDEERPYTDDELNDFEKSLEHAEQARDSQKDEDHDKQATTAKRTNL	85	36
CjPP1	MSRNITISGKGKRRRQYAAKRRGQYEAVRGRILTSNVDNQNKELVKIRTENDDTVVIERNRYGTYKVQDNNFIPDYYVTWKRVRVS	86	58
CjPP2	MGTSGAVSYKLIDKDISLVIYWKVTNTLTLSTKNMFFVQIEKRVSISPENLEQMLKNIDDK	61	17
CjPP3	MPAQLGFKGFLVREEGQESAVLVLGLKTKELQQNLLKTVPAGKQIPKVLINKFGKIIESGRVLPVDIEKSKTNELLPTMMERQQP	85	52
CjPP4[§]	MYMHKKSGNTYAFYPYEYKEQDLKVDVSQNGTVVTTTFTIANLNTTTDEATYTYHLSHTEDGRTSPTGTALTVLVPTVITSPNPKI	86	80
CjPP5	MLTTGMSDDVDGLRAEKDRLASDASSWQNKYNTIQKNYENISLEKQRLEERLASMQKTINHLENTNQGLSNDKNKLFHDFESAKRKIHDLQSDLDSCRLGKDNLQAENDVLLKRVAQLEAEIDIQSKSNIEDYDDSSSYAVLQAAYKRSQERENELQSELLNAYKINARLDNENKALLENLLLS	184	66

[§] partial *N*-terminal sequence available;
* – mature toxins have the same precursor protein.

Table S4: Polypeptide sequences of proteins from sea anemone *C. japonicus* venom. E-value is calculated by pBLAST for the closest homologs. Specie name from which the homolog is isolated and superfamily accessory by UniProt/CD are listed. (VP – venom protein; PVP – possible venom protein; LPVP – low possibility venom protein). If signal sequence is found, length is denoted for both precursor and mature proteins (for mature proteins numbers are shown in parentheses).

Protein	Sequence	Len.	Superfamily	Homology	E-value	Ident. %	Organism	Type
CjVP1	LNLVKIGVSACSDVGKEEARDASSTSMV<br>NKLVVRGGTHKTRAELTDERDKTTIANF<br>LQEGVTDPSPIAYNFIPIWEILQTRFIGTQS<br>MAKVFNLEQFYKGYLHFGCPYEETSNGK<br>VLQKFVLSKDSDPDAPTYKCMLGPTGCR<br>VNNDCHYKPAFWCACAGESCVRYKDVK<br>LNSGTTKQEAYAYTSESPGWDWQGCKLS<br>FFECKCKEPRSNWEESWTGESDDGLRDIH<br>SVLAMERSSYSDSQNNCVKNTTSSDIL	254	MAC/<br>Perforin domain	Toxin AvTX-60A	2E-79	51%	*Actineria villosa*	Sea anemone
CjVP2	GYKTSCEMLCLVFWYHQPIENRTTLLLK<br>RTMNRLIILCLFAVMFGTTIAFPMEDISKD<br>EAIETISQRELEVPDKKSVTGTVIEGADLN<br>AVILDKVLTEIGGYLREIAIGVDNESGGE<br>WTAKNVYYFSGTSHARLPETVPYNKAFL<br>YSARKESGPVAAGAVGVLTYGLSDGNTL<br>AVLFSVPYDYNLYSNWWNVKVYSGNKR<br>ADFNMYHDLYYTANPFKGDDGWHKRLL<br>GNGLEMKGIMNSASHATLQIRVSCVSCY<br>NY	257	Sea anemone cytotoxic protein	Urticinatoxin	4E-90	63%	*Urticina crassicornis*	Sea anemone
CjVP3	LTTSRGGNGQQRTCTSSFWYLTSASSRDS<br>PEHQSVPLHARMNSGLVLTGVVGVLTYG<br>LRDGNTLAVLFSVPYSYFLTSNYWNVKV<br>YSGNKRADYNMFYDLYYKDKPFKGDDG<br>WHERSLGNGLKMKGIMNSAGHAKIANPC<br>ELCELL	146	Sea anemone cytotoxic protein	Bandaporin	8E-37	58%	*Anthopleura asiatica*	Sea anemone
CjVP4	EDYLHFFSNKSMCYSSVGKQGGKQKISIG<br>DGCERKGTVIHEMLHSIGFIHEQSRPDRD<br>KYVKVVVWRNIKKKLTHNFKKYPRYLVD<br>NLGVNYDFDSVLHYHNKAFTINGGDTLV<br>DKADPSRKFGQRISFSKRNIEQVNKLYDC<br>DVDPHVADDVIYE	155	Zinc-dependent metalloprotease	Zinc metalloprotei nase nas-13	1E-39	47%	*Caenorhabditis elegans*	Nematode
CjVP5	MTWLYTKKMIRREKEDDDMVMMKRQP<br>YSDSYYHWPKTDGVVNVPYTTSGVDNT<br>VLQRAIEVFNAKTCVRFVPRSSQANYVTF<br>RSGGSCHAAVGNQKSGQQFCTLSGGCAA<br>SKGKVVHEMMHVLSFLHEQCRSDRDKYI<br>TIHMDRIINGAAVNFQVVAGKDLGIPYDC<br>DSILQYKGTAFPSTAGQKTITSTYCSALGN<br>DDTRLSLLDYKRVNTLYGCDPNPDYKYY<br>KVRVVTDSSTISGSGTDCFVYLTLHGSSR<br>SSTEYELDLDVAGSNLFEAGSIDTFNYMA<br>YRNDLGSIEGISIRMVSNGRGEAWWPTTF<br>TVTDPATSAAVTFTNNKWLSPSSTYGRSN<br>FPKQ	345	Zinc-dependent metalloprotease	Zinc metalloprotei nase nas-14	2E-23	36%	*Caenorhabditis elegans*	Nematode
CjPVP1	KDFLERKMKAFSLVVISFVLLGGFAASSA<br>EESDLDITEDQNEPAVAKRSVIQFYELIRR<br>YTKRNPKDFLGYGCWCGYGGKGKPLDR<br>LDRCCRTHDWCYNKLTHRGHCGRYEVY<br>RKNYYYNGLTCYTGWWSSRCGRGLCRC<br>DVAAVKCFKRSHFNNRYKNWNKRKCX	166<br>(137)	PLA2_like superfamily	AcPLA2	1E-49	51%	*Adamsia palliata*	Sea anemone

CjPVP2	RTRLVRCWKWTCRLWVDDSCGTGRNPF DYNGYGCYCGFNGYGIPVDDVDRCCQT HDRCYGRVTSSGACPFSVATYIFPYTTTD CTNCKPASYYWFYGRCRHALCQCDSESV KCFRRSKYNSRYANYDKSKC	131	PLA2_like superfamily	CgPLA2	5E-22	46%	*Condylactis gigantea*	Sea anemone
CjPVP3	CMRGCILTYMRTSHDDTSLFTRINMNHL KCKMFMIISAMILCVSLHCEAKYDWETK TFTKEVNRFVFPGTKWCGKGNNAMSFD DLGIHNETDLCCREHDHCPTYILPFNRRY GIMNFYPSHINHCSCEKRLHAMSMERHQ SCSYSCX	147	PLA2_like superfamily	Phospholipas e A2 isozymes PA3A/PA3B/ PA5	5E-19	44%	*Heloderma suspectum*	Lizard
CjPVP4	GGLPRSRHSKMDLYGILLAILFFCSSLYLV ALIEDDESTLRQEEKISQIQEDGHSVQKRN LVQFALMIKCATGRNPLDYNNYGNWCG SGGHGVPVDGVDRCCRAHDLCYGRYGH CRPKWKTYKHRTRSRTSGWRRRRRSSGR KRSYHRCRISCRNRLGLCSRAVCECDRV AALCFARKRYNKKHKKNWWG	190	PLA2_like superfamily	UcPLA2	6E-37	45%	*Urticina crassicornis*	Sea anemone
CjPVP5	LYEGDIKLSEDQDEMLEFPSKKGKEKLSS NRAYTAGREEKRVWFGGVIPYVIDCSLK NMPGLVAVVKRAMKEWESKTCVLFVER DQEEDYVEIFRGTHCFSTVGRAKGKKVLS VGHGCEYHNVMLHELGHVIGYWHEQSR PDRDEHIRVLWKHVMPGWESTFLKLNW KAVDTMRLPYDYSSIMHYPFNAFSKNTR KRTIVPLKPVIARPYKRLSQLDILKTNIMY SCSNSAEDNEVDVDQQHSSVAALRRKKE QWW	256	Zinc-dependent metalloprotease	Zinc metalloprotei nase nas-6	2E-45	39%	*Caenorhabditis elegans*	Nematode
CjPVP6	SIQCYTSVIHCPSHASLERQGTVHHVVPR TSQTDYISFFSGPGCYSSVGRIGRKQRISIG PGCDSMGIIAHEIGHALGFWHEQSRPDRD QHVKILWNNIQPGNEVQFIKVKATNSLGV PYDLASLMHYGPKAFSINNLDTIQSLDGS TNFGQRNGLSDKDIEQAKLLYCQGINVCL HKDKSAYCPGWGAAGYCDSSSIHYKFM GKKCKRT	210	Zinc-dependent metalloprotease	Zinc metalloprotei nase nas-13	4E-37	46%	*Caenorhabditis elegans*	Nematode
CjPVP7	RLKRAAMSNRARRWMGADQRPLIPYVIE PSNYPARSQILKAMKHWTQNVPCLRFQR RTSQRSYLSFYAGGGCFSHVGRVGGRQRI SIGRGCERHLSVVVHEIGHALGFWHEQSR PDRDRYVKIYWNNILPQFKYAFHRYADG RINSLGVPYDYDSVMHYDSKAFGQGRTT ITRINGDTRLGNRRGLSTMDIKQARLLYC GSGGTLPTRPPRPATRPPTGGCPHKDLSS WCSYWSSQGYCSSSSQHYPYVSVNCKKT CFCKKP	262	Zinc-dependent metalloprotease	Zinc metalloprotei nase nas-6	7E-44	34%	*Caenorhabditis elegans*	Nematode
CjPVP8	PGGKVPYVFGRVSNRVKSAFKQAIADYN KYTCVRVVPRSSETSYIVVVSAGGCWSSL GKSGGMQKLSLGRGCEHKGTAIHELMHA LGFFHEQSRRDRDSHITIHWNNIMPGREN NFKKYRHGKADTLNEPYDYGSIMHYPRK AFSKNGKETIATKTSGVSIGQRRTFSVIDI RQINKLYKCKGIVNPTKMPTTKPGPCVDF QAGCKIWAKE	211	Zinc-dependent metalloprotease	Zinc metalloprotei nase nas-15	7E-54	48%	*Caenorhabditis elegans*	Nematode
CjPVP9	VDNTVLQRAIEVFNAKTCVRFVPRSSQA NYVTFSGGSCHAAVGNQKSGQQTCDLTS GCAASKGKVVHEMMHILGFLHEQCRSDR DSYITIHKDRIISGASANFDVISGTDQGIPY DCDSILQYKGTAFAATAGQKTITSTYCSA LGNDDTRLSLLDYKRVNTLYGCDPNLX	171	Zinc-dependent metalloprotease	Zinc metalloprotei nase nas-30	2E-25	39%	*Caenorhabditis elegans*	Nematode

CjPVP10	VMMDDMVVHKKMLRREKDDDLVMMK<br>RQAFNDDYYRWPKTDGVVNVPYTTSGV<br>DNTVLQRAIEVFNAKTCVRFVPRSSQAN<br>YVTFRSGGSCHAAVGNQKSGQQFCTLSG<br>GCAASKGKVVHEMMHVLSFLHEQCRSD<br>RDKYITIHMDRIINGAAVNFQVVAGKDLG<br>IPYDCDSILQYKGTAFPSTAGQKTITSTYC<br>SALGNDDTRLSLLDYKRVNTLYGCDPNP<br>DYKYYKVRVDD	233	Zinc-dependent metalloprotease	Zinc metalloproteinase nas-14	3E-24	36%	*Caenorhabditis elegans*	Nematode
CjPVP11	MAMHYSILVFALFIGACWAGAIKELKKR<br>EVDLEKSNLTDSCLDAAQKRAGDCYKNS<br>CYTFHINGKTWKKNRKACQVKGGDLVS<br>MEDDCEWLYITSKIQKLGPPLGDEWHIGL<br>MKKGKNNWQWVSGARLSVRHWRKFQP<br>SGGNELYGVMVTEFPSPCKECGTFNDVP<br>DHLKRGYICEKSG	179 (160)	C-type lectin	hypothetical protein NEMVEDRAFT_v1g248367	1E-09	34%	*Nematostella vectensis*	Sea anemone
CjPVP12	MRLSLVILVLAITVCFPALSSACCDQGWL<br>KFGSHCYFVNHDKQGLANWEDAKRTCG<br>FLGGHLVDIRDEAEWMFVKRLLQPGPTIN<br>GVAKQYMIGMSDAGSERQWKRFDGSRV<br>TFLKWSNGEPNGGTNENCGGIVANTGLY<br>NDGPCNFKYGRFICKKTEAS	160 (138)	C-type lectin	Predicted protein	3E-21	38%	*Nematostella vectensis*	Sea anemone
CjPVP13	YFFNHDKQGLAKWENAKQICSFLGGHLV<br>DIRDEAEWMFVKSLLQPAGAYLIGIGDAG<br>SEGKWKYTGDGSLVKFFKWKSGEPNNA<br>GINEHCGTMYSDTGLYNDDGCDYKRGRF<br>ICKKAE	118	C-type lectin	Neurocan core protein-like	6E-14	38%	*Hydra vulgaris*	Fresh-water polyp
CjLPVP1	MREGFVEEVDSPRENVTYIVNILTGNEWG<br>AGTDSNIHFTLYGSAGESEQQTIENEGSTT<br>FECGMTDKYKFECPYLGMLTKLRIGHDN<br>TGWGPAWYLENVTVIGPKTEENVVFPCN<br>RWLATNADDGMIERELVREGMFGDNIIIE<br>GLTDGVRGEPLGLESGEIKDENITASSHAE<br>ATPPSSARLNNTVGWCAKEAEGSWLQVD<br>LENLYCITSVATQGNPSGNQDYVKKYKL<br>QYSQDGHKWTNHEENGNQIFDANVDSST<br>VKTNDLQKVIISRYIRFCPVEWYCWPCM<br>RVEVYGAPLQEGGTEVRTVSYVTEQIVVS<br>QEGKEVESVPDNEEEKHKAEDAIQFILSQ<br>TGDKRKDIEVQKKVIDDEMQKLEDERKE<br>REKRKEEERRQMNEEERKKDEEEQRKRE<br>EEQKNKELEEEERRKKVELEVKKIEDEHQ<br>LRLAELDRKIKEDEEKHRKEIEEQKKLDE<br>EKLKELDDEMKKDEEMIKQHEETRLKYE<br>EKMKELQLAGIIKTENLTKTIDGIPRDDGV<br>ETITITRQIVVESSVEDDKKDDDLDGKEK<br>NKEERAREREDKIKKYRKTKKNVKQKRK<br>ISMKLCCRRE	583	Coagulation factor 5/8 C-terminal domain	Venom prothrombin activator omicarin-C non-catalytic subunit	1E-20	40%	*Oxyuranus microlepidotus*	Snake
CjLPVP2	AGAAEGSDVEQEALVLHNKFRTIHNAPE<br>MKLNAEMSQSAASYAAQIANQGSLSHSS<br>SEARDNNGENLSYGCSSSKGQTPTEAISN<br>WYNEVCNPGYSFGSESMGGAGHFTQLV<br>WKESVELGFGKADVDQNGMKCSYYVGR<br>YKKAGNMGGEFAKNVVKGNFDQSYCST<br>VKRSDGLFKRAHTGIAEVRKRQ	188	Cysteine-rich secretory protein family	Cysteine-rich venom protein helothermine	4E-08	35%	*Heloderma horridum horridum*	Snake
CjLPVP3	MRGCAWLDLCFFFGLVFFAGPVYTRILQ<br>VFIPDSKSTVPHSALQAHNKYRAMHHSPL<br>LNWSEPLADQAQAIVDTMARGGSFSAGQ<br>RNKAVNLGQNLAKLAGFMTCDDAGEIAT<br>NLWYSQAKNYSYSDPRLNADTDTFTQVV<br>WKTSKEIGVGCARSPNNQSGPVYIVALY<br>RPAGNIPRLLRGNVLSPGTKGADPDVYST<br>LFRRNYFRKPKISKTEMP	216	Cysteine-rich secretory protein family	Cysteine-rich venom protein catrin	0.005	40%	*Crotalus atrox*	Snake

Table S5: Sequences of oligonucleotides.

Name	Sequence 5'->3'
280-Bam	AGGATCCATGGGTGTGCCGTGCCGCTGCGATAGCGATGGTCCGAG
280-Hind	TATAAGCTTCATTTCTTTGCAGCATTCATACGCAATGTT
280-1	TGCGATAGCGATGGTCCGAGCGTGCATGGCAACACCCTGAGCGGC
280-2	GCAACACCCTGAGCGGCACCGTGTGGGTGGGCAGCTGCGCGTCAG
280-3	GTTGTATTCATCGTTGCATTTATGCCAGCCTGACGCGCAGCTGCCCA
280-4	GCAGCATTCATACGCAATGTTGTATTCATCGTTGCATTTATG
280-MBam	AGGATCCATGGGTGTGCCGAGCCGCAGCGATAGCGATGGTCCGAG
280-MHind	TATAAGCTTCATTTCTTTACTGCTTTCATACGCAATGTT
280-M1	AGCGATAGCGATGGTCCGAGCGTGCATGGCAACACCCTGAGCGGC
280-M2	GCAACACCCTGAGCGGCACCGTGTGGGTGGGCAGCAGCGCGTCAG
280-M3	GTTGTATTCATCGTTGCTTTTATGCCAGCCTGACGCGCTGCTGCCCA
280-M4	ACTGCTTTCATACGCAATGTTGTATTCATCGTTGCTTTTAT
1c-Bam	TGGATCCATGCCGTGCCGTTGCGAATCCGATGGTCCTCCTCGTC
1c-Hind	TATAAGCTTCATCTCTTTGCAGCAGGTGGAAATCGCATTGATG
1c-1	CGATGGTCCTCCTCGTCAGAATAATGCGCTGTCAGGCACCACCT
1c-2	GCTGTCAGGCACCACCTTCTATGTGGTGGGCTGCAATAAAGC
1c-3	GATGTAACGGCATTTATTCCAGCCTGCTTTATTGCAGCCCACC
1c-4	CAGGTGGAAATCGCATTGATGTAACGGCATTTATTCCA
L7-Bam	AGGATCCATGGGCTGCGGCTGCCATACCGAATGCTCCTTAC
L7-Hind	ATAAAGCTTCATCCAGGAGCAACGGCAACGCAGACCGCA
L7-1	CATACCGAATGCTCCTTACAGTGCTCCTTTTCCGGCTGCGGCT
L7-2	ACGGCAACGCAGACCGCACACATAGCCGCAGCCGGAAAAG
L8-Bam	TGGATCCATGTGCGATCCGGACAAACGTGATTCCGTGTG
L8-Hind	TATAAGCTTCATAAACAGATCCACGCAGCACACTTCTTTGCCA
L8-1	GACAAACGTGATTCCGTGTGCAAAGATGTGTGCGGTCTGCTG
L8-2	GATGTGTGCGGTCTGCTGGATATTGGCACCGAGAATGGCGA
L8-3	GCAGCACACTTCTTTGCCAGGACATTCGCCATTCTCGGTGCC

Table S6: Table shows recombinant peptides sequence and average masses of peptides with different C-terminal modifications. C- termini methionine residues (green), amino acid substitutions (red), cysteine residues (blue).

name	sequence	C-terminal methionine* (Da)	C-terminal homoserine * (Da)	C-terminal homoserine* lactone (Da)
CjTL8	CDPDKRDSVCKDVCGLLDIGTENGECPGKEVCCVDLFM	4107.60	4071.60	4053.60
AnmTx Cj 1c-1	PCRCESDGPPRQNNALSGTTFYVVGCNKAGWNKCRYINAISTCCKEM	5181.87	5151.87	5133.87
CjTL7	GCGCHTECSLQCSFSGCGYVCGLRCRCSWM	3229.78	3199.78	3181.78
native δ-actitoxin -Cgg1a	GVPCRCDSDGPSVHGNTLSGTVWVGSCASGWHKCNDEYNIAYECCKEM	5161.66	5131.66	5113.66
mutant δ-actitoxin -Cgg1a	GVPSRSDSDGPSVHGNTLSGTVWVGSSASGWHKSNDEYNIAYESSKEM	5071.27	5041.27	5023.27

* - average mass if all cysteines are oxidized

Table S7: AnmTX Cj 1c-1 peptide toxicity. **N**- the number of experimental animals.

Acute toxicity action of AnmTX Cj 1c-1 on shrimps*				
**Dose, µg/g**	**Lethality in 3h, %**	**Paralysis, %**	**Comments**	**N**
1	0	16,7	Paralysis starts 10-20 sec after injection, full remission may be seen after 12 h	6
5	0	16,7		6
8	16,7	33,3		6
10	66,7	100	Paralysis is immediate, remission may be seen after 12 h	6
20	66,7	100		6
30	100	100	Paralysis is immediate, with no remission	6
**Action of the negative control - Bovine Serum Albumin (BSA) solution on shrimps****				
13	did not happen	did not happen	-	6
260	did not happen	did not happen	-	6
**Action of the negative control - physiological salt solution on shrimps****				
-	did not happen	did not happen	-	6
**Acute toxicity action of AnmTX Cj 1c-1 on insect larvae*****				
1	0	0	Negative control - physiological salt solution	18
30	50	100		18

* - Volume of the injected solution 5 µl. **- Volume of the injected solution 1 µl. ***- Volume of the injected solution 2 µl.

Table S8: CjTL8 peptide toxicity. **N**- the number of experimental animals.

Acute toxicity action of CjTL8 on shrimps*				
**Dose, µg/g**	**Lethality in 3h, %**	**Paralysis, %**	**Comments**	**N**
1	0	100	Paralysis starts in 10-20 sec after injection; full remission may be seen after 12 h	6
2,7	50	100		6
3,5	66,7	100		6
4	80	100		6
10	100	100		6
30	100	100	Paralysis is immediate, with no remission	6
**Action of the negative control - Bovine Serum Albumin (BSA) solution on shrimps****				
13	did not happen	did not happen	-	6
260	did not happen	did not happen	-	6
**Action of the negative control - physiological salt solution on shrimps****				
-	did not happen	did not happen	-	6
**A cute toxicity action of CjTL8 on insect larvae*****				
1	0	0	Paralysis or any other effect is not observed; Negative control - physiological salt solution	18
30	0	0		18

* - Volume of the injected solution 5 µl. **- Volume of the injected solution 1 µl. ***- Volume of the injected solution 2 µl.

Table S9: CjTL7 peptide toxicity. **N**- the number of experimental animals.

<table>
<tr><th colspan="5">Acute toxicity action of CjTL7 on shrimps*</th></tr>
<tr><th>Dose, µg/g</th><th>Lethality in 3h, %</th><th>Paralysis, %</th><th>Comments</th><th>N</th></tr>
<tr><td>10</td><td>0</td><td>0</td><td>This injection induced intensive convulsive legs movements enduring for 12h. After that shrimps were behaving normally, their movements were undistinguishable from control's. No lethal effect is observed in 24 h.</td><td>6</td></tr>
<tr><td>25</td><td>0</td><td>100</td><td rowspan="4">Short-term paralysis, then active moving around vessel for 15 sec. Then convulsive movements with the paralysis frontal legs for 5-10 min. After that shrimps are behaving normally, they are undistinguishable from controls. This 'calm' behavior lasts for approximately 15 min, and then shrimps start to move extremely quick around the vessel with short periods when they stay put. During short 'stay put' periods (20-30 sec) intensive frontal legs convulsions are observed. Shrimps tend to exhibit this behavior for approximately 1 h, and then they finally calm down and stay motionless but alive in the bottom of the vessel. No lethal effect is observed during 24 hours after injection.</td><td>6</td></tr>
<tr><td>50</td><td>0</td><td>100</td><td>6</td></tr>
<tr><td>100</td><td>0</td><td>100</td><td>6</td></tr>
<tr><td>150</td><td>0</td><td>100</td><td>6</td></tr>
<tr><th colspan="5">Action of the negative control - Bovine Serum Albumin (BSA) solution on shrimps**</th></tr>
<tr><td>50</td><td>did not happen</td><td>did not happen</td><td>-</td><td>6</td></tr>
<tr><td>100</td><td>did not happen</td><td>did not happen</td><td>-</td><td>6</td></tr>
<tr><th colspan="5">Action of the negative control - physiological salt solution on shrimps**</th></tr>
<tr><td>-</td><td>did not happen</td><td>did not happen</td><td>-</td><td>6</td></tr>
<tr><th colspan="5">Acute toxicity action of CjTL7 on insect larvae***</th></tr>
<tr><td>10</td><td>0</td><td>0</td><td rowspan="2">Paralysis or any other effect is not observed; Negative control - physiological salt solution</td><td>18</td></tr>
<tr><td>150</td><td>0</td><td>0</td><td>18</td></tr>
</table>

* - Volume of the injected solution 5 µl. **- Volume of the injected solution 1 µl. ***- Volume of the injected solution 2 µl.

Table S10: Insect-toxicity tests. **N**- the number of experimental animals. Volume of the injected solution 2 µl.

Dose (µg/g)	Lethality (%), AnmTx Cj 1c-1	N	Lethality (%), ω-Tbo-IT1	N	Lethality (%), δ-Actitoxin-Cgg1a	N
5	16,66667	18	16,66667	18	100	18
20	16,66667	18	50	18	100	18
30	50	18	100	18	100	18
70	100	18	100	18	100	18

Supplementary Text:

Discussion of some of the most important classes of proteins, which are identified only by transcriptomics.

Proteins that are deduced from transcriptomic data only may be as interesting as those that are validated by proteomics. Full-length sequences of these venom proteins are presented in Table S3.

At first, 4 phospholipase sequences (CjPVP1 – CjPVP4) were present in both specimens. Amino acid sequences of phospholipases have a high degree of homology with known components of sea anemone venoms such as CgPLA2 (*Condylactis gigantea*), AcPLA2 (*Adamsia palliata*), and UcPLA2 (*Urticina crassicornis*). One of the sequences we found is similar to Phospholipase A2 from the gila monster (*Heloderma suspectum*, a venomous lizard). All the listed phospholipases belong to the PLA2 family. Representatives of this family are components of venoms of different animals, including Arthropoda (scorpions[75–77], *hymenoptera*[78–80]), Gastropoda (cone snails),[81] sea anemones,[81–86] and Reptilia (snakes[87–93] and lizards[87]). These molecules facilitate the functions of prey capture and digestion and defence against other animals. However, phospholipases can also induce multiple types of responses, such as irritation and systemic envenomation in humans.[94] For phospholipases PLA2 (detected in different natural sources), quite different types of functional activities are revealed: anticoagulatory, myotoxicity, lytic activity towards plasma membrane of the affected muscle cell,[95] inflammatory, antioxidant, anti-inflammatory, phagocytic function regulation, CNS regulation, membrane trafficking, and leukocyte chemotaxis. Therefore, the activities of PLA2 phospholipases are diverse and might be important for the overall actions of the venom.

Secondly, C-lectin-like proteins (CjPVP11 – CjPVP13), which may play a wide spectrum of roles in multicellular organisms, are also found. Immune response,[96–98] intercellular interactions,[99] and endocytosis and apoptosis[100] are the processes in which C-lectin-like proteins participate, and they are promising for anti-cancer therapy.[101] It is known that C-lectin-like proteins may be important components of snake venoms.[102–105] Calcium-dependent lectins may induce hemaglutination, edemas, hyperpermeability of vessels, and reduction of arterial tension.[106,107] We have deduced several proteins homologs of lectins, which may potentially be acting components of venoms.

Supplementary materials and methods:

Reads assembly and annotation

Basic filters recommended for qualitative analysis of Ion Torrent PGM were applied to the raw reads, and the adapters used for cDNA synthesis were trimmed. For *de novo* transcriptome assembly, Newbler (Roche Diagnostics, Basel, Switzerland) was used in cDNA assembly mode. CLC Genomic Workbench (CLCbio a Qiagen Company, Aarhus, Denmark) was used in default mode. Reads with lengths less than 30 bp were not used for assembly. To group and annotate all the unigenes, we used local BLAST (BlastX algorithm threshold value of $e = 1 \times 10^{-6}$, matrix BLOSUM-62) against the protein databases NR and SWISS-PROT. Blast2GO was used to analyse gene ontology and to functionally annotate contigs and isotigs. Contig taxonomic distribution visualisation (KEGG; KOG/EGGNOG classifications) was conducted using MEGAN5 software. To identify ORF, mark up, and annotate, TransDecoder (Broad Institute; CSIRO) was used.

Other computational tools

Cutadapt v1.9 was used for trimming, which eliminated adapter sequences used for cDNA synthesis. Prinseq lite v.0.20.4 was used for reads quality and length trimming. ClustalW2 and MUSCLE algorithms integrated into package UGENE v.1.16 (Unipro, Novosibirsk, Russia) were used to perform multiple alignment construction and visualisation. FigTree v.1.4.2 was used to visualise phylogenetic trees. Translation of selected nucleotide sequences to amino acid by all 6 frames was done with Nucleotide Sequence Translation EMBOSS Transeq/EMBOSS Sixpack (http://www.ebi.ac.uk/Tools/st/) online tool. Batch Web CD-Search Tool (http://www.ncbi.nlm.nih.gov/Structure/bwrpsb/bwrpsb.cgi) was used to detect conservative domains. Stand-alone software package SignalP 4.1 Server was used to predict signal sequences.

Generation of the DNA fragments encoding toxin and plasmid construction for the expression in E.coli.

The DNA fragments encoding new toxins (AnmTx Cj 1c-1, CjTL7, CjTL8), positive and negative controls (native δ-actitoxin-Cgg1a and his mutant) designed for subsequent expression in *E. coli* were generated by enzyme-mediated extension of synthetic oligonucleotides. To this end, we prepared the three mixtures of the oligonucleotides: from 1c-1 to 1c-4 for AnmTx Cj 1c-1, from L8-1 to L8-3 for CjTL8, L7-1 and L7-2 for CjTL7 (table S5). For controls we prepared the two mixtures of the oligonucleotides: from 280-1 to 280-4 for native δ-actitoxin-Cgg1a, from 280-M1 to 280-M4 for mutant δ-actitoxin-Cgg1a (Supplementary table 5). The concentration of each oligonucleotide was 10 μM. One microliter of the each mixture was transferred into a tube filled with 20 μl of a solution containing 1x PCR-buffer, the mixture of dNTPs (the concentration of each dNTP was 0.2 mM) and 0.25 units of Taq-polymerase. The reaction mixture was incubated as follows: 95°C – 10 sec, 60°C - 10 sec, 72°C – 15 sec, for a total of 5 cycles. The obtained products of the reaction were used as matrices for PCR with the 1C-Bam and 1C-Hind oligonucleotides in case of AnmTx Cj 1c-1, L7-Bam and L7-Hind in case of CjTL7, L8-Bam and L8-Hind in case of CjTL8, 280-Bam and 280-Hind in case of native δ-actitoxin-Cgg1a, 280-MBam and 280-MHind in case of mutant δ-actitoxin-Cgg1a. The DNA fragments, encoding toxins was introduced into the pET-32a(+) plasmid (Novagen, USA) using the BamHI and HindIII restriction sites. Thus, we constructed five plasmids encoding new toxins AnmTx Cj 1c-1, CjTL7, CjTL8 and controls native/mutant δ -actitoxin-Cgg1a fused with *E. coli* thioredoxin and the C-terminal 6HisTag (see Figure S4 for pET32-anem1C-1 plasmid).

Expression and purification

We used laboratory fermenter Brunswick BioFlo 110 Fermentor/Bioreactor (Fisher Scientific) a volume of 5 liters. The target plasmids were introduced into *E. coli* BL21 (DE3) gold by heat-shock transformation and seeded on Petri dishes with LB agar and ampicillin. Pre-cultures of *E. coli* were grown overnight in 300 mL LB2x medium supplemented with ampicillin (100 μg/mL) at 37°C with shaking at 180 rpm. Overnight cultures were transferred to 3L of fresh medium LB2x and were grown at 37ºC until an OD value of 0.6-0.8 at 600 nm was reached. Isopropyl-β-D-thiogalactopyranoside (IPTG) was added to a final concentration of 1 mM, cultures were further grown 4 hours at 37 ºC. Cells were harvested by centrifugation and resuspended in 150 mL of distilled water. The cells were disrupted by gentle sonication, the precipitate was separated by centrifugation at 15000g 15min. To the lysate was added 1/7 part of 8x buffer A (final concentration - 20 mM monosodium phosphate, 0.5 M NaCl, 10 mM imidazole, pH 7,5), re-centrifuged at 15000g 15min. The resulting solutions were placed in a chromatographic column (XK-16/20; GE Healthcare, USA) filled with 10 ml of sorbent Ni Sepharose High Performance (GE Healthcare, USA) and equilibrated with the same buffer. After application, the columns were washed with at least 50 ml of starting buffer and then washed again with 20 mM Na-phosphate buffer (pH 7.5) containing 25 mM imidazole, and 0.5 M NaCl. Subsequently, the protein was eluted with 20 mM Na-phosphate buffer (pH 7.5) containing, 0.5 M NaCl, and 500 mM imidazole. The flow rate was 2 ml/min. The inspection of the process and the collection of the fractions were performed via measurements of the eluate absorbance at 280 nm. The target fused proteins were purified using a chromatograph AKTA FPLC (GE Healthcare, USA).

Recombinant toxins production

Fusion proteins (see Figure S5 and S6) cleavage for target toxins release was performed using direct cyanogen bromide cleavage protocol (with omission of the desalting step).[62] All fusion protein solutions were diluted to a concentration of 0.1 mg/ml. HCl to a final concentration of 0.2 M and CNBr with a molar ratio to a fusion protein of 600:1 were added. Lowering cleavage temperature along with increasing time of the reaction was found to be beneficial for obtaining particular toxins of this research work. Cleavage reaction was performed at 14ºC during 20-22 h, whereupon cyanogen bromide was evaporated using Savant SpeedVac SVC 100H centrifugal evaporator.

For isolation of recombinant toxins from reaction mixture RP-HPLC method was utilised. Chromatographic separation for each toxin was conducted stepwise. Preliminary rough purification on semi-preparative Phenomenex Jupiter C_5 (21.20x250 mm) 300Å 10μm column was performed using a linear gradient of acetonitrile (0.1% v/v TFA containing buffers) at a flow rate of 5 ml/min. For the final purification either Grace Davison Discovery Sciences Vydac HPLC column #218TP54 (C_{18}, 4.6x250 mm,

5µm), or Phenomenex Synergy Polar-RP column (4.6x250 mm) 80Å 4µm was used. For both columns, a linear gradient of acetonitrile (0.1% v/v TFA containing buffers) at a flow rate of 1 ml/min was exploited. In all the cases, concentration of acetonitrile had been raised from 0% to 60% for 60 min. Completeness of toxins purification and toxins identities to deduced sequences were examined using Bruker Ultraflex II-MALDI TOF/TOF mass-spectrometry (see Figure S7-10) and ESI-Q mass-spectrometry on Shimadzu LCMS-2020 (see Figure S11).

Supplementary references:

75. Rajendra, W., Armugam, A. & Jeyaseelan, K. Toxins in anti-nociception and anti-inflammation. *Toxicon* **44,** 1–17 (2004).

76. Farsky, S. H. P., Antunes, E. & Mello, S. B. V. Pro and antiinflammatory properties of toxins from animal venoms. *Curr. Drug Targets. Inflamm. Allergy* **4,** 401–11 (2005).

77. Valentin, E. & Lambeau, G. What can venom phospholipases A(2) tell us about the functional diversity of mammalian secreted phospholipases A(2)? *Biochimie* **82,** 815–31

78. Habermann, E. Bee and wasp venoms. *Science* **177,** 314–22 (1972).

79. Müller, U. R. Hymenoptera venom proteins and peptides for diagnosis and treatment of venom allergic patients. *Inflamm. Allergy Drug Targets* **10,** 420–8 (2011).

80. Monteiro, M. C., Romão, P. R. T. & Soares, A. M. Pharmacological perspectives of wasp venom. *Protein Pept. Lett.* **16,** 944–52 (2009).

81. Nevalainen, T. J., Morgado, I. & Cardoso, J. C. R. Identification of novel phospholipase A2 group IX members in metazoans. *Biochimie* **95,** 1534–43 (2013).

82. Nevalainen, T. J. *et al.* Phospholipase A2 in cnidaria. *Comp. Biochem. Physiol. B. Biochem. Mol. Biol.* **139,** 731–5 (2004).

83. Landucci, E. C. T. *et al.* Purification and inflammatory edema induced by two PLA2 (Anch TX-I and Anch TX-II) from sea anemone Anthothoe chilensis (Actiniaria: Sagartiidae). *Comp. Biochem. Physiol. B. Biochem. Mol. Biol.* **161,** 170–7 (2012).

84. Talvinen, K. A. & Nevalainen, T. J. Cloning of a novel phospholipase A2 from the cnidarian Adamsia carciniopados. *Comp. Biochem. Physiol. B. Biochem. Mol. Biol.* **132,** 571–8 (2002).

85. Grotendorst, G. R. & Hessinger, D. A. Enzymatic characterization of the major phospholipase A2 component of sea anemone (Aiptasia pallida) nematocyst venom. *Toxicon* **38,** 931–43 (2000).

86. del Monte-Martínez, A. *et al.* Improved purification and enzymatic properties of a mixture of Sticholysin I and II: isotoxins with hemolytic and phospholipase A(2) activities from the sea anemone Stichodactyla helianthus. *Protein Expr. Purif.* **95,** 57–66 (2014).

87. Kudryavtsev, D. *et al.* Natural compounds interacting with nicotinic acetylcholine receptors: from low-molecular weight ones to peptides and proteins. *Toxins (Basel).* **7,** 1683–701 (2015).

88. Calvete, J. J. Proteomics in Venom Research: a Focus on PLA2 Molecules. *Acta Chim. Slov.* **58,** 629–37 (2011).

89. Kordiš, D. Evolution of phospholipase A2 toxins in venomous animals. *Acta Chim. Slov.* **58,** 638–46 (2011).

90. Lomonte, B. & Gutiérrez, J. M. Phospholipases A2 from viperidae snake venoms: how do they induce skeletal muscle damage? *Acta Chim. Slov.* **58,** 647–59 (2011).

91. Faure, G. & Saul, F. Structural and Functional Characterization of Anticoagulant, FXa-binding Viperidae Snake Venom Phospholipases A2. *Acta Chim. Slov.* **58,** 671–7 (2011).

92. Carvalho, B. M. A. *et al.* Snake Venom PLA2s Inhibitors Isolated from Brazilian Plants: Synthetic and Natural Molecules. *Biomed Res. Int.* **2013,** 1–8 (2013).

93. Fernandes, C. A. H., Borges, R. J., Lomonte, B. & Fontes, M. R. M. A structure-based proposal for a comprehensive myotoxic mechanism of phospholipase A2-like proteins from viperid snake venoms. *Biochim. Biophys. Acta* **1844,** 2265–2276 (2014).

94. Harris, J. & Scott-Davey, T. Secreted Phospholipases A2 of Snake Venoms: Effects on the Peripheral Neuromuscular System with Comments on the Role of Phospholipases A2 in Disorders of the CNS and Their Uses in Industry. *Toxins (Basel).* **5,** 2533–2571 (2013).

95. Ownby, C. L., Selistre de Araujo, H. S., White, S. P. & Fletcher, J. E. Lysine 49 phospholipase A2 proteins. *Toxicon* **37,** 411–45 (1999).

96. Geijtenbeek, T. B. H., van Vliet, S. J., Engering, A., ’t Hart, B. A. & van Kooyk, Y. Self- and nonself-recognition by C-type lectins on dendritic cells. *Annu. Rev. Immunol.* **22,** 33–54 (2004).

97. Dambuza, I. M. & Brown, G. D. C-type lectins in immunity: recent developments. *Curr. Opin. Immunol.* **32,** 21–27 (2015).

98. Weis, W. I., Taylor, M. E. & Drickamer, K. The C-type lectin superfamily in the immune system. *Immunol. Rev.* **163,** 19–34 (1998).

99. Cummings, R. D. & McEver, R. P. C-type Lectins. (2009).

100. Kim, M. *et al.* Lectin-induced apoptosis of tumour cells. *Glycobiology* **3,** 447–53 (1993).

101. Yau, T., Dan, X., Ng, C. C. W. & Ng, T. B. Lectins with potential for anti-cancer therapy. *Molecules* **20,** 3791–810 (2015).

102. Ogawa, T., Chijiwa, T., Oda-Ueda, N. & Ohno, M. Molecular diversity and accelerated evolution of C-type lectin-like proteins from snake venom. *Toxicon* **45,** 1–14 (2005).

103. Takeya, H. *et al.* Coagulation factor X activating enzyme from Russell’s viper venom (RVV-X). A novel metalloproteinase with disintegrin (platelet aggregation inhibitor)-like and C-type lectin-like domains. *J. Biol. Chem.* **267,** 14109–17 (1992).

104. Hirabayashi, J., Kusunoki, T. & Kasai, K. Complete primary structure of a galactose-specific lectin from the venom of the rattlesnake Crotalus atrox. Homologies with Ca2(+)-dependent-type lectins. *J. Biol. Chem.* **266,** 2320–6 (1991).

105. Ogilvie, M. L., Byl, J. W. & Gartner, T. K. Platelet-aggregation is stimulated by lactose-inhibitable snake venom lectins. *Thromb. Haemost.* **62,** 704–7 (1989).

106. McEver, R. P. Selectins: initiators of leukocyte adhesion and signaling at the vascular wall. *Cardiovasc. Res.* **107,** 331–9 (2015).

107. Choi, K.-M. *et al.* Functional characterisation and expression analysis of recombinant serum amyloid P isoform 1 (RbSAP1) from rock bream (Oplegnathus fasciatus). *Fish Shellfish Immunol.* **45,** 277–85 (2015).
